# Toward Compostable Packaging: Biodegradable Polymer Blends with Biobased Components for Household Chemical Bottle-Layer Applications

**DOI:** 10.1021/acsomega.5c10689

**Published:** 2026-07-07

**Authors:** Sebastian Kowalczyk, Matylda Szewczyk-Łagodzińska, Maciej Dębowski, Anna Iuliano, Monika Truskolaska, Maria Krywult-Pawlik, Gabriela Jędrzejczak, Natalia Grochowska, Andrzej Plichta

**Affiliations:** † Faculty of Chemistry, Chair of Polymer Chemistry and Technology, 49566 Warsaw University of Technology, Noakowskiego 3, 00-664 Warsaw, Poland; ‡ GRUPA INCO S.A., Wspólna 25, 00-519 Warsaw, Poland

## Abstract

Biodegradable polyester compositions were developed as model systems for potential application in multilayer bottles for household chemicals. Polylactide (PLA) was modified with an epoxy-based chain extender (ECE) to adjust chain topology and molar mass to extrusion blow molding processing and with a carbodiimide hydrolysis stabilizer (HS) to ensure long-term durability. Additional blends with poly­(butylene adipate-*co*-terephthalate) (PBAT), poly­(3-hydroxybutyrate-*co*-3-hydroxyvalerate) (PHBV), and inorganic fillers (CaCO_3_, talc) were prepared to tailor toughness-strength-elasticity balance and processing behavior. Gel permeation chromatography (GPC) revealed that ECE significantly increased weight-average molar mass (*M*_w_) and generated multimodal distributions, while HS introduced low-molar-mass fractions (≈11–50 kg mol^–1^), broadening dispersity but providing a capacity for future hydrolytic stabilization. Mechanical testing showed that ECE improved tensile strength and stiffness, HS enhanced ductility and impact resistance, and PBAT incorporation introduced cavitation-assisted plastic deformation, visible in SEM. Thermal analysis confirmed sufficient stability for melt processing (*T*_5_% > 250 °C), with talc inducing crack deflection and rougher fracture surfaces compared to CaCO_3_, which often caused particle pull-out and local voiding. Rheological measurements highlighted that CaCO_3_ increased melt flow through interfacial lubrication effects, whereas ECE reduced flow due to branching. Together, the results demonstrate complementary roles of ECE, HS, and fillers in balancing molar mass, thermal robustness, mechanical performance, processability, and morphology, thereby establishing design principles for compostable high-performance packaging materials.

## Graphical abstract

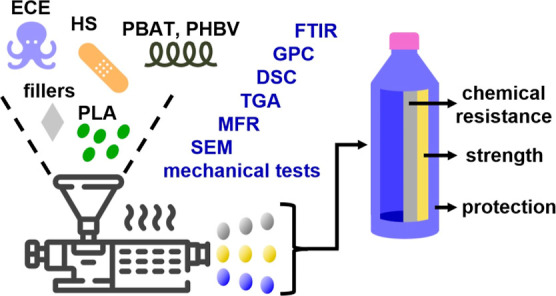

## Introduction

Our society depends on plastics. Our houses are built with their help, and parts of our cars, planes, boats, and trains are partially made from them. They find many uses in medicine (syringes, blood bags, heart valves, etc.) and military (helmets, tank parts). They are in our electronic devices, but first of all, they are widely used for packaging.^1^ This versatility of purposes can be attributed to the ease of polymer-based material manufacturing and processing, as well as their favorable physical and chemical properties.^2^ For example, they are characterized by low density; therefore, using plastic instead of glass or metal packaging reduces transportation costs, energy consumption, and greenhouse gas emissions. Replacing metals with plastic composites in car and plane parts results in a significant decrease in fuel consumption. Moreover, polymer materials used in packaging can allow for prolonged food storage, which is invaluable due to the geographical spread of our food production system.^3^

Unfortunately, plastics have some disadvantages. Polymer synthesis requires excessive amounts of energy, and if it comes from nonrenewable sources, it leads to high emissions of CO_2_. This phenomenon is exacerbated if we consider incineration with energy recovery as an end-of-life application of polymer materials. However, if we replace conventional, petroleum-based polymers with bioderived ones, we can minimize the negative environmental impact. Poly­(lactic acid) (PLA) is produced using multistep (bio)­technological processes comprising lactic acid (LA), lactide, and polymer synthesis. The resources of LA are usually carbohydrates from plants, e.g., corn, sugar cane, and tapioca, which capture CO_2_ in the process of photosynthesis.^4^ For example, the synthesis using nonrenewable energy sources and incineration of 1 kg of poly­(ethylene terephthalate) (PET) leads to the emission of 4.93 kg of CO_2_ and that of polystyrene (PS) to 5.98 kg, while 1 kg of bioderived PLA leads to only 3.84 kg of CO_2_ emitted; hence, replacing PET and PS with PLA can cut greenhouse gas emissions by 20% and over 30%, respectively.^5^ Furthermore, using renewable energy sources to produce PLA can lead to a closed-loop CO_2_ cycle, and if it is recycled instead of being incinerated, it can act as a greenhouse gas sink.^6^ However, only 9% of plastic waste generated worldwide up to 2019 got recycled, while the rest got incinerated or accumulated in landfills and the environment.^7,8^ The buildup of plastics in the environment leads to the emergence of harmful microplastics (plastics less than 5 mm in size), which can pollute the Earth for tens or even hundreds of years if they are made of nonbiodegradable polymers.^9,10^ Those concerns are especially alarming due to the large share of plastic that is produced. In 2022 alone, 400.3 Mt of plastic was produced, with 90.5% of this volume being virgin, conventional plastics and around 99.5% being nonbiodegradable and/or bioderived.^11,12^ The European Union (EU) treats this environmental threat very seriously and addresses it in its legislation, which is crucial as it produces high amounts of plastic waste, 26 million tonnes annually, and it is predicted that by 2030 this number will increase even by 46% if no action is taken.^13,14^ The situation is even more severe in the US, as they produce around 35 million tonnes of plastic waste annually while having a smaller population than the EU.^15^

Since plastic is an inseparable part of our lives but may negatively affect the environment, different measures are being taken to mitigate its impact. Those actions include forcing societies to recycle plastic and using bioplastics, which include bioderived and/or biodegradable plastics. It is important to note that there are different types of bioplastics. Some are biodegradable, some are compostable, some are marine degradable, and some do not decompose easily.^16^ The word ‘biodegradable’ is very general and includes a lot of different decomposing conditions. For example, all PLA, poly­(butylene adipate-*co*-terephthalate) (PBAT), and polyhydroxybutyrate (PHB) are often called biodegradable, but PLA needs to be industrially composted to be broken down into small components in a reasonable time (weeks), while PBAT can biodegrade in soil and home compost, whereas PHB can even biodegrade in landfill, freshwater, and marine environment.^17–19^ Additionally, biopolymers can be biobased (derived at least partially from degradable sources of carbon) and/or petroleum-based. To illustrate, polycaprolactone (PCL) and PBAT are biopolymers as they can biodegrade, even though they are fossil fuel-based, as well as bioderived polyethylene (bio-PE), which is considered nondegradable, while PLA and PHB are both bioderived and biodegradable.^20–22^ Therefore, since the definition of bioplastics is so broad, it is difficult to talk about their advantages and disadvantages in general, and it can be preferable to use more precise language, especially since companies tend to use such terms as “bioplastic”, “biobased”, “biodegradable”, etc. to greenwash their products.^14^

Bioplastics come with their costs and benefits. Producing bioderived plastics often requires repurposing of land that was used for food production, using fertilizers and pesticides, and chemically processing organic matter into plastic, consuming a lot of energy.^16,23^ On the other hand, they can help us cut our greenhouse gas emissions. Biodegradable plastics can help to solve the microplastic pollution problem. One of the most popular biopolymers is PLA. Furthermore, PLA has a high tensile strength (∼55 MPa), similar to PS (∼55 MPa) and higher than PET (∼50 MPa).^24,25^ Therefore, PLA is being highly researched and is gaining popularity as a packaging material.^26^ However, PLA can also be characterized by low impact strength (oriented PLA has half of the impact strength of PET in a free-falling dart test), and since it is an aliphatic polyester, it is prone to hydrolytic degradation.^24,27^ This hydrolytic degradation is a great problem when PLA is exposed to high temperatures, like during processing and mechanical recycling, or high- or low-pH solutions, which limits the use of PLA as a packaging material for household chemicals and cleaning products.^28^ Furthermore, PLA is also prone to thermo- and photo-oxidative degradation pathways.^27^ Therefore, neat PLA cannot be used reliably, and it requires the addition of stabilizers in its products. This raises costs of production and causes additional problems, like the migration of additives to the surface of the product or even their sweating. Finally, PLA has a melt flow index (MFI), which is much too high for extrusion blow molding (EBM) and can be excessive for injection blow molding (IBM) too. This restricts the usage of PLA as a packaging material, as those methods are popularly used to produce plastic bottles, with the EBM method allowing for layered bottle production. However, PLA single-layer bottles can be produced using the injection stretch blow molding (ISBM) method, and they are used as water, edible oil, or short shelf life milk containers.^4,29^ Another strategy that was used to produce bottles for storing light-sensitive foods is to make PLA-containing blends.^30^ This allows lowering the MFI of the compositions and using the EBM technique. PLA bottles are already produced and utilized for storing foods; however, they are yet to be applied as household chemical containers, especially due to the aforementioned PLA’s pH sensitivity. So far mostly petroleum-based, nonbiodegradable plastics, like high-density polyethylene (HDPE), low-density polyethylene (LDPE), and polypropylene (PP), are used for these applications.^31,32^

The aim of the research was to develop a material concept for the production of packaging that is intended for the storage and transport of household chemicals. The expected result was to produce compositions that meet the requirements of modern consumers, with sufficient durability, strength, and functionality. These materials should come from renewable sources to the greatest extent possible and at the same time be biodegradable in industrial composting conditions. The concept was based on the use of PLA as one of the most easily available biodegradable polymers. However, this polymer was subjected to numerous chemical and physical modifications to eliminate its material defects. The obtained polymer blends were characterized structurally, and their mechanical and thermal properties were determined.

## Experimental Section

### Materials

INGEO 2003D (PLA, Nature Works), ECOFLEX F Blend C1200 (PBAT, BASF), ENMAT Y1000P (poly­(3-hydroxybutyrate-*co*-3-hydroxyvalerate), PHBV, TianAn Biologic Materials), KRITILEN FILLER PL776 (contains 60 wt % of CaCO_3_ dispersed in a PLA carrier, Plastika Kritis), KRITILEN NC PL830 (contains 60 wt % of talc dispersed in a PLA carrier, Plastika Kritis), Joncryl ADR 4468 (epoxy chain extender, ECE, BASF), and Stabaxol P 100 (hydrolysis stabilizer, HS, Lanxess) were used without purification.

### Processing Method

#### Obtaining Ready-Made Polymer Mixtures Using the Extrusion Method

A twin-screw extruder from Krauss Maffei Berstorff Getriebeeinheit ZE25A.580UTXi was used to obtain polymer mixtures. A corotating screw system with a rotational speed of 40 to 60 rpm was used. A pressure of approximately 50 MPa was applied, the temperatures in subsequent zones were set in the temperature range of 25–180 °C, and the screw torque ranged from 60 to 80%. The material was dosed into the hopper in an amount of 3–4 kg, collected in the form of a string, which was then introduced into the granulator (granulation speed was between 4 and 6 m/min), and the finished mixture was collected in the form of granules.

#### Obtaining Samples Used to Measure Mechanical Properties by Injection Molding

The resulting mixtures were injected on an Arburg Allrounder 170S 180–30 hydraulic injection molding machine. After loading the granulate into the hopper and setting the appropriate process parameters, the material was placed in a heated cylinder where the plastic was plasticized. The temperatures of subsequent zones ranged from 160 to 190 °C, the dosing volume was 7.3 cm^3^ at a pressure of 1300 bar. The pressing time was 9 s, the pressure was 1100–800 bar, and the cooling lasted 40 s at a pressure of 75 bar. The obtained shapes consisted of 60 “dog-bone” tensile test specimen and 130 Charpy impact and three-point flexural test specimen bars, which were used for further research.

#### Measurements

Differential scanning calorimetry (DSC) was taken with a TA Instruments Q2000 calorimeter. Polymer samples (in the amount of about 10 mg) were subjected to time-tested temperature profiles: heating I from 25 to 200 °C at a rate of 10 °C min^–1^, cooling from 200 °C to −80 °C at a rate of 20 °C min^–1^, and heating II from −80 to 200 °C at a rate of 20 °C min^–1^. Thermogravimetric analysis (TGA) was made with a TA Instrument SDTG600. Polymer samples in the amount of approximately 10 mg were heated in the temperature range from 10 to 1000 °C with a heating rate of 10 °C min^–1^. The process was carried out in an inert gas atmosphere. Fourier-transform infrared spectroscopy (FTIR) spectra were recorded on a Thermo Scientific Nicolet iS5 spectrometer. The molar mass and molar mass distribution were determined by gel permeation chromatography (GPC) on a Viscotek system comprising GPCmax and TDA 305 [triple detection array (TDA): RI, IV, LS] equipped with DVB Jordi gel column(s) (linear, mixed bed) in DCM as an eluent at 30 °C at a flow rate of 1.0 mL min^–1^. Conventional calibration with the ReadyCal set of 12 narrow polystyrene standards of *M*_p_ in the range of 400–2,000,000 g mol^–1^ was used. Tensile strength and three-point flexural tests were conducted by the Instron 5566 Universal Testing Machine, equipped with a 10 kN measuring head. In case tensile tests, the machine was equipped with self-tightening roller tensile grips; for 30 samples in the shape of “dog bone”, tests were performed at room temperature at a running rate of 50 mm min^–1^. However, flexural tests for 30 bar-shaped samples placed horizontally on two supports spaced 32 mm apart and bent at a central point were performed at room temperature at a running rate of 50 mm min^–1^. Data was processed with BlueHill2 software. Impact strength measurements were carried out at room temperature using a Zwick HIT50 Plus device equipped with a pendulum with a nominal energy of 7.5 J for 30 bar-shaped samples in a configuration without or with a V-notch. Surface topography studies were performed by scanning electron microscope (SEM) analysis using Hitachi SU8000. The *melt flow rates* of the compositions were determined using a Zwick/Roell Mflow apparatus, with measurements conducted at 190 °C under a load of 2.16 kg.

## Results and Discussion

### Manufacturing Biopolymer Blend with Additives

The manufactured materials are dedicated to obtaining compostable bottles for household chemicals. Such bottles must meet many requirements: good mechanical resistance to static stresses (e.g., tensile and bending strength) and dynamic stresses (impact strength), resistance to water and media with different pH, compostability, and rheological properties appropriate to the selected packaging production method. It seems that it might be very tough or even impossible to find and fabricate one material which fulfills all above the requirements. Therefore, the idea is to utilize the EBM technique for the production of bottles, which gives the opportunity to manufacture multilayered containers, so each compostable layer would bring various sets of required properties. For that reason, the set of materials of different compositions and properties is developed in this work to choose the best potential candidates for three layers of the bottle’s wall: (a) outer barrier for contact with the user and external environment, (b) middle layer responsible for appropriate mechanical properties, and (c) internal barrier for contact with various chemical media.

The quite simple compositions of the first series of 11 materials are presented in Table 1. The base polymer for all compositions is PLA, which is the best-known and developed bioderived and biodegradable polymer, but it does not meet all of the criteria given above. Therefore, it was mixed with one or two (inert or reactive) components using a twin-screw corotating extruder (equipped with efficient mixing zones) in order to point out their influence on certain properties. There were additives of three groups used: (a) elastic biodegradable polyesters, (b) mineral fillers, and (c) reactive epoxy chain extender. Aliphatic-aromatic copolymer PBAT and bacterial aliphatic polyester PHBV from group (a) were used to study their influence on elasticity and impact strength. On the other hand, the chalk and talc from (b) were applied in order to check their impact on mechanical properties, crystallization ability, and rheology. Finally, ECE, from (c) category, was introduced in a low amount into some of the single- or two-component systems to improve mechanical and rheological parameters and hydrolytic stabilization.

**Table 1:** Compositions of the First Series of Mixtures (without HS)

	weight fraction (wt %)					
material	PLA	PBAT	PHBV	CaCO_3_	talc	ECE
M1	100	-	-	-	-	
M2	99.5	-	-	-	-	0.5
M3	99	-	-	-	-	1
M4	95	5	-	-	-	-
M5	95	-	5	-	-	-
M6	94.5	-	5	-	-	0.5
M7	90	-	-	10	-	
M8	89.5	-	-	10	-	0.5
M9	89.25	-	-	10	-	0.75
M10	93.75	-	-	-	5	1.25
M11	88.75	-	-	-	10	1.25

While the results revealed some trends (see further discussions) the more complex systems M12–M19 (comprising 3–6 components) were prepared, consisting of PLA, the additives from the above groups (a)–(c), and the carbodiimide moieties-based hydrolysis stabilizer HS, which acts as a chain extender or a rejoining agent as well (Table 2).

**Table 2:** Compositions of the Second Series of Mixtures (with HS)

	weight fraction (wt %)^a^				
material	PLA	PBAT	PHBV	CaCO_3_	talc
M12	97.25	-	-	-	-
M13	87.25	-	-	10	-
M14	92.25	-	-	-	5
M15	87.25	-	-	-	10
M16	77.25	10	-	10	-
M17	67.25	20	-	-	10
M18	77.25	-	10	10	-
M19	57.25	15	15	-	10

a In all mixtures listed, 0.75 wt % of ECE and 2 wt % of HS were present.

In all those compositions, reactive ingredients ECE and HS were used at constant amounts of 0.75 and 2 wt %, respectively. It is important that the mechanism of HS acting (also at elevated temperature) is based on the nonequilibrium esterification of hydroxyl and carboxyl groups with water molecule uptake, leading to an extended polyester chain and urea derivative as a side product. Generally, all the polyesters used in this work are hydroxyl or carboxyl end-capped, so HS action in the case of them leads to the formation of longer homo- or copolymers (in case there is more than one kind of polyester present in the mixture). It means that (multi)­segmental copolymers might be obtained as well, which can play a role of compatibilizers in case of mixing of two (or more) thermodynamically immiscible polyesters (e.g., PLA and PBAT).^33^ It works differently with ECE, which contains several epoxy groups per molecule, which can undergo ring-opening reactions with carboxyl groups and, less preferably, with hydroxyl ones, forming ester or ether linkages, respectively, and newly formed hydroxyl groups. Because ECE generates a branched polymer population of grafted topology, however, similar to HS, blocks of different polyesters may be grafted, too, which formally gives copolymers. Moreover, the hydroxyl groups generated during ECE ring opening exhibit high reactivity toward the adduct formed by the reaction of HS with the terminal carboxyl groups of polyesters but also with other epoxy moieties in ECE or with polyester chains while taking part in the segment exchange process. Therefore, the presence of ECE and HS in the systems studied causes the formation of miscellaneous chemical structures that may benefit material properties (Figure 1).

**Figure 1:**
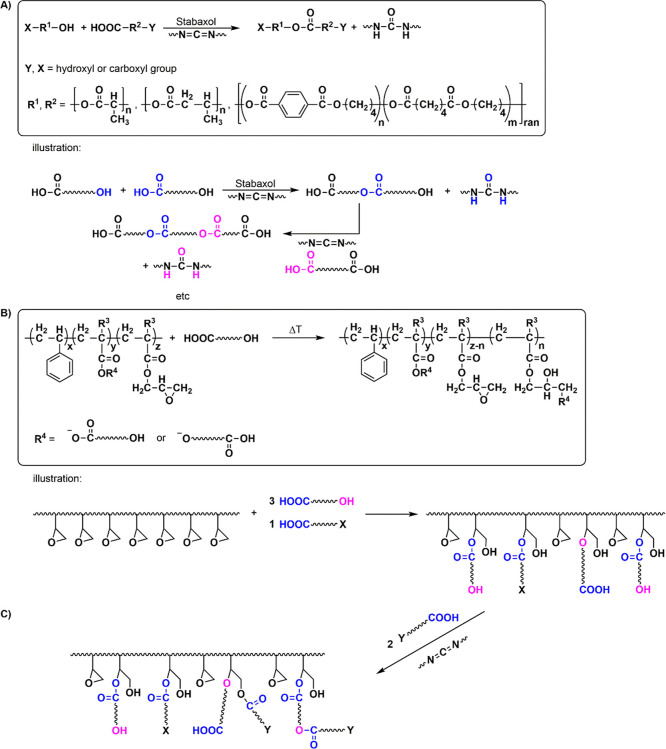
Scheme of the chemical action of the additives used - ester formation via (A) PLA chains coupling in the presence of HS, (B) PLA chain extension with ECE, and (C) PLA chains coupling onto a hydroxyl-ECE derivative in the presence of HS.

The complex chemical reactions described above result in changes of molar mass values and distributions, including broadness and shape (multimodality) of GPC traces. It is influenced as well by the presence in the thermally processed mixture of various polymers characterized with different molar mass distributions and by some side reactions, e.g., hydrolysis and thermal (radical) chain destruction. To analyze obtained molar mass distributions, *M*_n_, *M*_w_, and *D̵* were calculated for the full distribution of each sample, whereas *M*_p_ values were determined for all fractions which show separate local maximum. The molar mass distribution for individual compositions is shown in Figure 2, while the detailed values are shown in Table S1. Taking into account that some molar mass distributions (of the main population) were positively skewed, exhibiting additional inflection points, which indicate overlapping molar mass fractions, these distributions were subjected to mathematical processing involving numerical fitting of two or three Gaussian subdistributions approximately composing the original curve. The subdistributions enabled us to estimate the peak values *M*_p_^fit^ of overlapped fractions (Figures S1–S10). When analyzing GPC results and traces, one can notice that neat PLA slightly reduces its molar masses after processing (M1) due to some polymer degradation; however, introducing ECE into the system at 0.5 wt % (M2) and 1 wt % (M3) causes, respectively, the formation of the additional high-molar-mass fraction of *M*_p_^fit^ 524 kg mol^–1^ and shifts the complete distribution toward high molar mass values (*M*_p_ 245 kg mol^–1^) with a significant branched fraction of *M*_p_^fit^ 920 kg mol^–1^. These phenomena are consistent with literature.^34^ The blend comprising PBAT (M4) reveals monomodal distribution with a bit lower *M*_w_ and *M*_p_ than M1 because of the lower molar mass of PBAT. Although PHBV (M5) shows reduction of molar masses with PLA and bimodal distribution, the introduction of ECE (M6) causes an increase in molar mass and the formation of multimodal distribution with three *M*_p_^fit^ of 129, 410, and 464 kg mol^–1^. It seems that ECE acts very efficaciously with two aliphatic polyesters, leading to highly branched structures. Application of chalk as a filler for PLA (M7) causes a decrease in molar masses, probably due to some side reactions catalyzed by Ca ions, which may be present in the system. However, the introduction of 0.5 wt % (M8) or 0.75 wt % (M9) of ECE counteracts this trend, and higher molar masses are observed. Similar findings are established for talc composites; however, in filled systems there is no multimodality even when as high as 1.25 wt % of ECE is used. In the case of the majority of samples containing HS (series M12-M19), the fraction is characterized by fitted *M*_p_ in the range of 11–50 kg mol^–1^ is present. Taking into account the molar mass parameters of HS, it seems that the population comes from HS used or its hydrated derivative. We checked out that both Stabaxol P100 and its (partially or fully) hydrated derivative formed the model reaction at esterification of acetic acid with ethanol and are soluble in the GPC eluent (Figure S11). The FTIR spectra of HS (Stabaxol P100) and its partial urea-like derivative are shown on Figure S12, together with spectra of compositions: M2 without HS and M12 containing HS. Although the spectra of HS and its derivative display distinct differences related to characteristic bands of the respective forms (e.g.,–N=C=N–, –NH–, and –NH–C­(=O)–NH–), these bands are not detected in the polymer compositions, presumably because of the insufficient HS content in the system. Therefore, it cannot be unambiguously determined whether HS in these mixtures is in its pure form or partially, or even completely, hydrated. In the system with copresence of ECE and HS, the much broader and multimodal distributions are observed. It is caused by the HS fraction mentioned above but also by chain-extension processes induced by ECE and followed by subsequent reactions of hydroxyl groups originating from epoxy ring opening in ECE with carboxyl end-groups of polyester further promoted by HS. The large *D̵* and multimodal molar mass distributions are obtained as a result. Surprisingly, the sample filled with chalk and stabilized with ECE and HS (M13) reveals much lower *M*_n_ and *M*_w_ than that one without HS. Paradoxically, the presence of HS polymer of low molar mass decreases the overall molar mass parameters of the sample, whereas one could expect that it should promote condensation processes, which are supposed to elevate the molar masses. In general, the presence of chalk in the compositions comprising additional polyesters other than PLA (M16, M18) diminishes the molar masses (most clearly seen for *M*_w_), while talc filler (M14, M15, M17, M19) allows them to reach higher values than for calcium carbonate. In summary, the presence of oligomeric HS in the systems results in some reduction of average molar masses and does not promote the formation of additional high-molar-mass fractions. Therefore, one can deduce that HS in majority remains unused in studied systems, ready to counteract the hydrolysis issue in the future.

**Figure 2:**
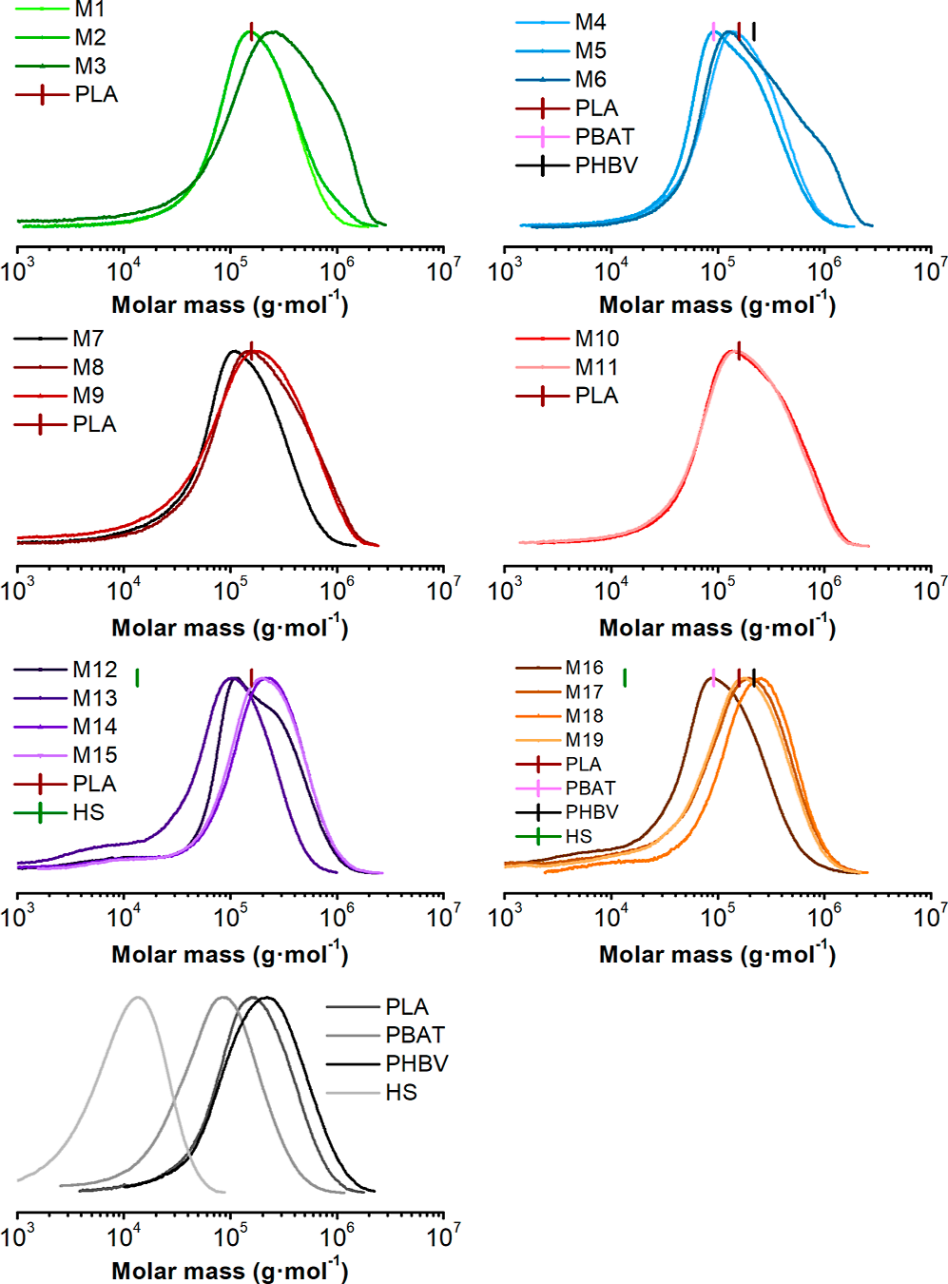
Distributions of molar masses of the obtained biopolymer mixtures.

### Mechanical Properties

During the first part of our studies, we have tested the impact of different polymeric and inorganic additives, as well as the epoxide chain extender (ECE) on the selected mechanical properties of PLA. A two-tailed student *t*-test (with α = 0.025) was used to check if the differences between tested materials are statistically significant. Tensile tests (Figure 3 and Table S2) showed that, within the estimation errors, the incorporation of 0.5–1.0 wt % of ECE (M2, M3) did not affect elasticity and ductility but improved tensile strength (σ_T_) of PLA (M1), especially for the system with the highest (1.0 wt %, M3) content of ECE. A very similar trend was also observed if a PLA composite loaded with CaCO_3_ was subjected to modification with ECE, although the achieved values of σ_T_ were lower than that of a neat PLA sample. These data indicate that the addition of 10 wt % of chalk (M7) substantially worsened the ability of PLA to effectively resist the uniaxially oriented mechanical stresses, and ECE (M8, M9) only minimized this negative effect. It is worth noting that the ECE modified composites utilizing talc exhibited better σ_T_ than those filled with chalk; however, statistically, their mechanical parameters did not differ from those of the unmodified polymer matrix. On the other hand, in the case of talc composites, a higher content of ECE was used than in chalk ones. Considering the mechanical properties of binary blends of PLA with either PBAT or PHBV, it is evident that the addition of these two polyesters led to a lower σ_TS_ of the resulting materials, as well as their increased elasticity and ductility compared with neat PLA (M1). Both blends, (M4—PLA/PBAT and M5—PLA/PHBV) exhibited lower mean values of Young’s modulus (*E*_T_) and higher values of elongation at break (ε_T_), but in the case of the former system, these changes were more pronounced.

**Figure 3:**
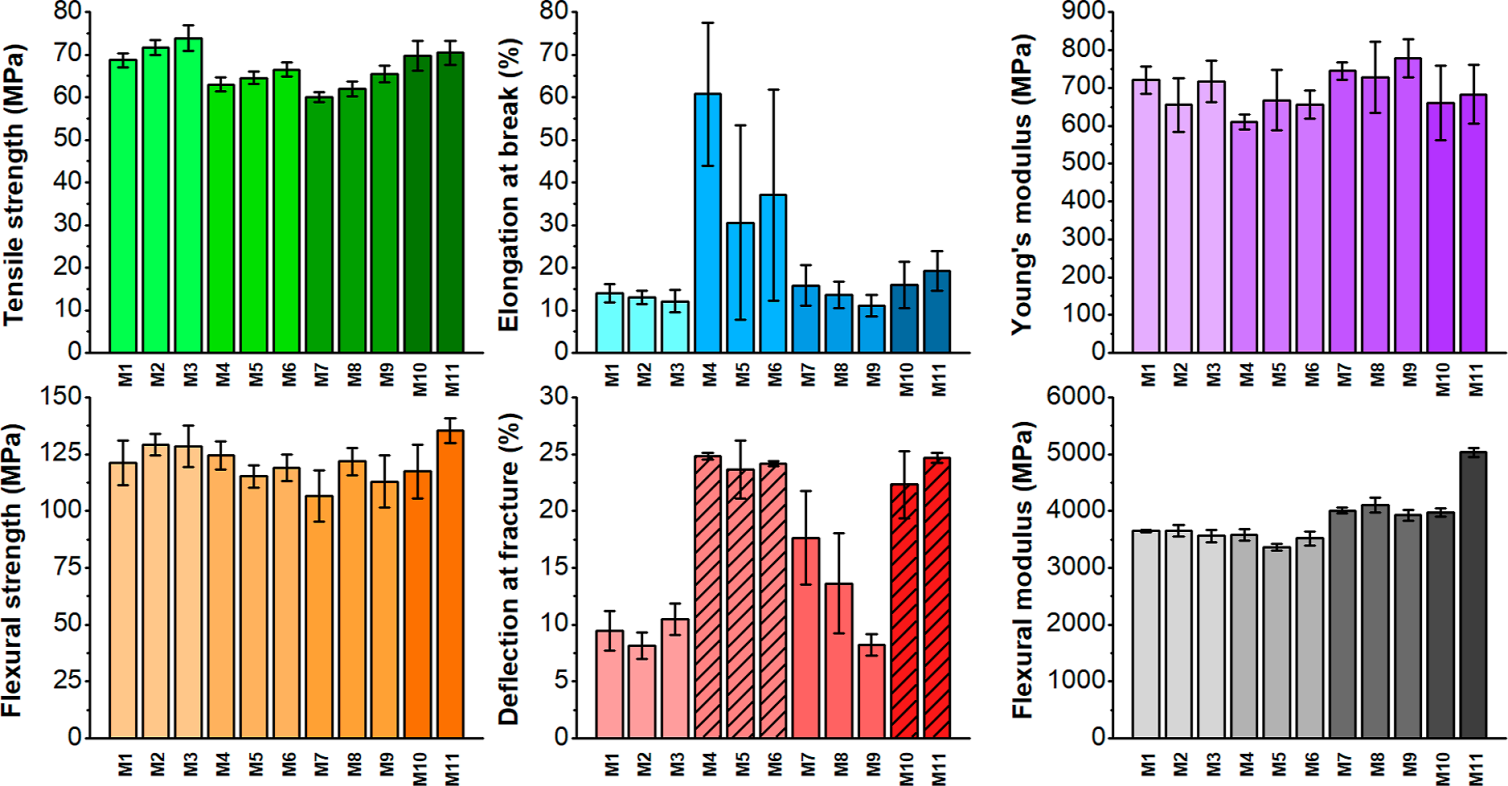
Mechanical properties (tensile and flexural tests) of the first group of biopolymer compositions (hatched bars represent some samples that remained unbroken during the test).

Flexural tests (Figure 3 and Table S3) showed that the incorporation of ECE or PBAT (M2–M4) had a minuscule impact on the elasticity of the resulting materials since the p-values of the observed changes (e.g., a decrease in flexural modulus (*E*_F_)) were only just above the threshold *p*-value—only in the case of the PLA/PHBV (M5) blend was this effect highly visible (its *E*_FT_ decreased by ca. 8% of the value characterizing neat PLA, M1). One can expect that the strength-enhancing effect of ECE also had an impact on the stiffening of the system, reducing the effect of PHBV. It is worth noting that the PLA/PBAT (M4) and PLA/PHBV (M5) blends or PLA composites loaded with talc (M10, M11) exhibited improved ductility compared to PLA; they did not fracture up to flexural deflection (ε_F_) of 25% (taken as the end point of the standard flexural test), whereas the other materials (neat PLA—M1 and PLA-based systems stabilized with ECE M2 and M3 or containing CaCO_3_—M7, M8, M9) showed ε_F_ between 8% and 18%. Interestingly, the presence of inorganic fillers (i.e., talc and chalk) significantly increased the stiffness of PLA during the flexural tests, as indicated by higher values of *E*_F_, especially in the case of a composite containing 10 wt % of talc and 1.25% of ECE whose *E*_F_ exceeded 5 GPa. As far as flexural strength (σ_F_) is considered, stabilization of PLA with ECE and its loading with 10 wt % of talc (M11) had a small positive effect on the value of this parameter: an increase in the mean value of σ_F_ for these materials was almost 15 MPa (i.e., 12.5% of the value determined for neat PLA).

ECE, PHBV, and mineral fillers proved to be very effective impact modifiers for PLA, as evidenced by the results of Charpy impact tests (Figure 4 and Table S4). From that point of view, inorganic compounds helped the most to effectively dissipate the energy of impact, with talc being the best—its composites with PLA, regardless of their composition (i.e., filler content—M10 and M11), exhibited more than 4 times higher Charpy impact strength than the unmodified PLA (M1). The impact strength is one of the crucial parameters for packaging materials, especially for household chemical bottles.

**Figure 4:**
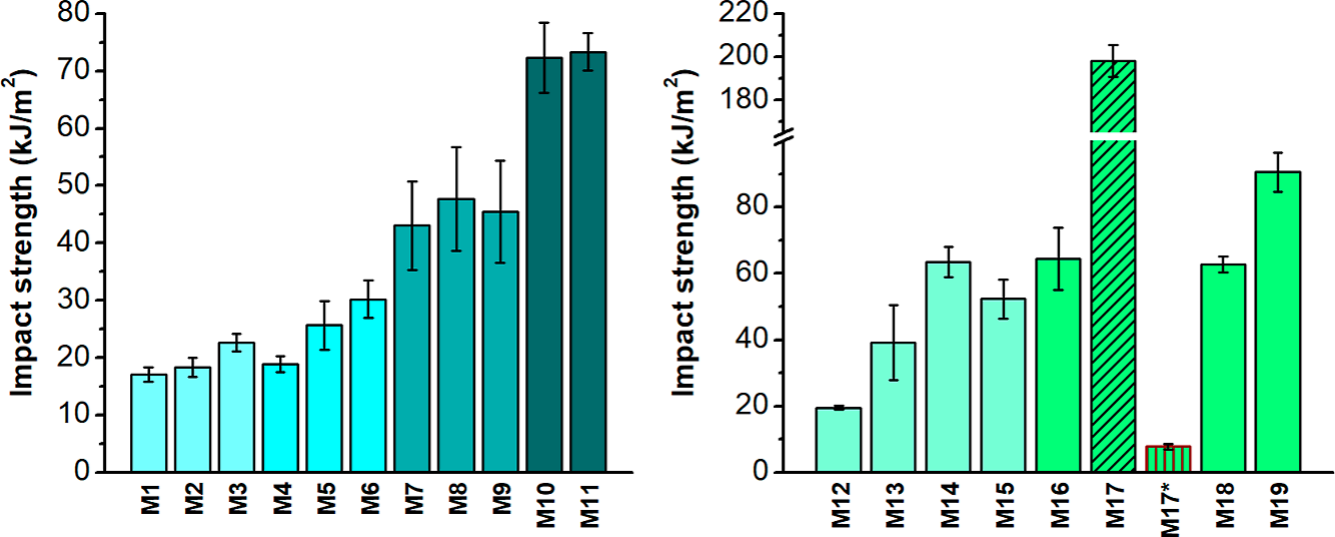
Charpy impact strength results for the mixtures (black hatched bars—sample did not fracture during testing; red hatched bars—measurement results for notched samples).

A second group of materials (M12–M19) subjected to mechanical tests consisted of the PLA-containing systems, chain-extended and stabilized with both 0.75 wt % of ECE and 2.0 wt % of HS (Stabaxol P-100), respectively. To these “base” polymeric matrices, different amounts of PBAT, PHBV, and/or inorganic fillers were added. The summary of tensile test parameters for this group is shown in Figure 5 and Table S2. The utilization of reactive additives only slightly increased σ_T_ of PLA, and, taking into account the previously discussed results obtained for PLA stabilized solely with ECE, one can assume that this mechanical strengthening was primarily due to the presence of epoxide-type stabilizer (M2, M3) rather than HS (M12). It looks reasonable, as the ECE is an active modifier of which molecules incorporate into the structure of the polymer matrix, combining polymer chains into branched structures comprising several arms (based on ECE functionality), whereas the HS is a stabilizing additive which only enables two or more chains of polymers to join into one linear chain. It should be noted that in the case of this type of hybrid stabilization of PLA, the incorporation of any other additives (e.g., inorganic fillers, such as chalk–M13 or talc–M14, M15, or their mixtures with biodegradable polyesters M16–M19) resulted in a decrease in σ_T_ of the material. In this context, the least negative impact was observed in composite systems modified with talc particles (M14 and M15), for which σ_T_ remained at a level close to neat PLA. Among this type of the investigated systems, those containing PBAT had by far the worst σ_T_, and its replacement with completely aliphatic PHBV only partially minimized this negative effect. Usually, the modification with PBAT led to an increase in both the susceptibility of the material to plastic deformations and its elastic properties. For example, the highest values of ε_TS_ and the lowest values of *E*_T_ were detected for the systems containing 10 wt % of talc and either 20 wt % of PBAT–M17 (remained undamaged under the measurement conditions) or 15 wt % PBAT and 15 wt % PHBV–M19.

**Figure 5:**
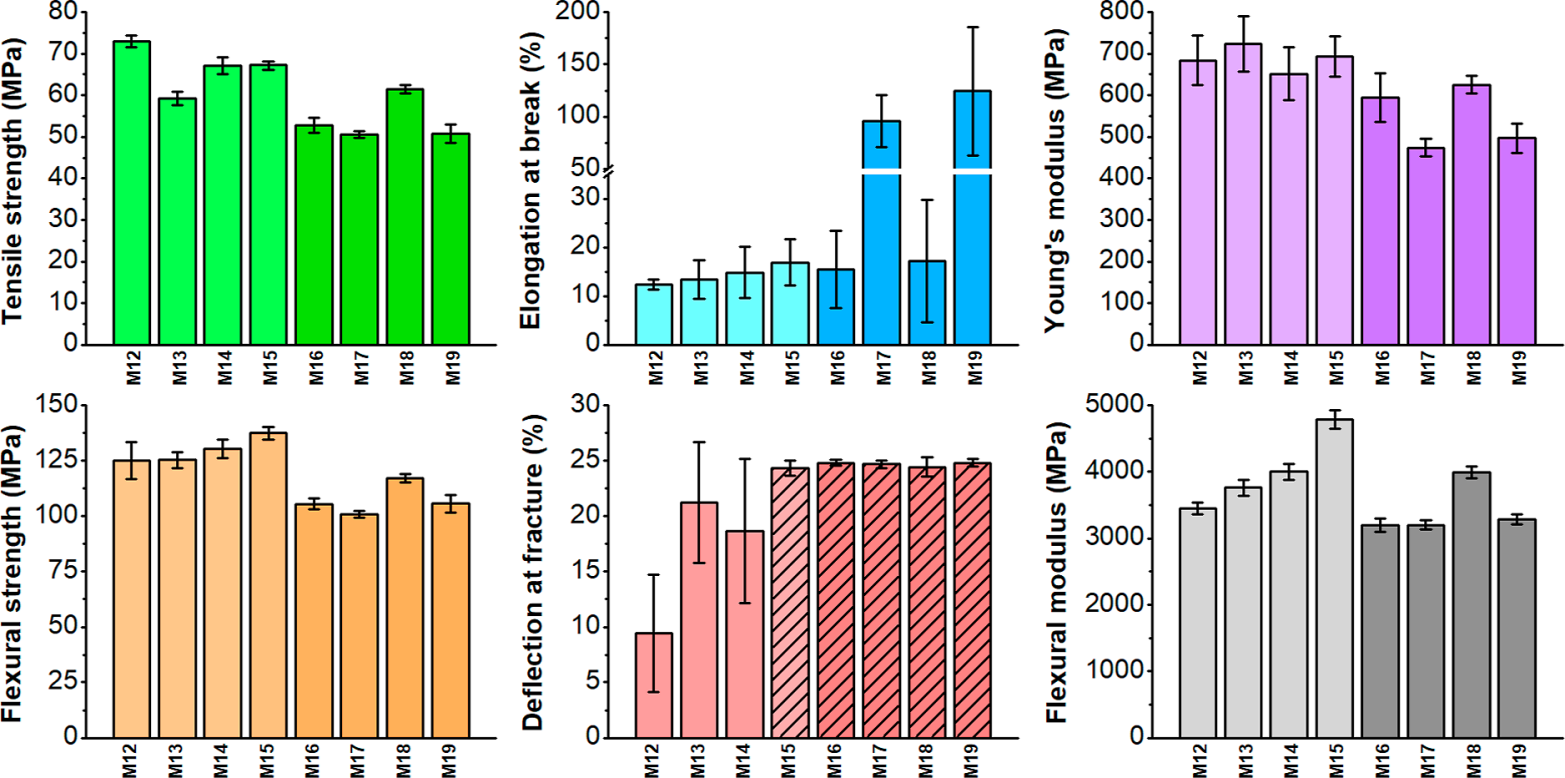
Mechanical properties (tensile and flexural tests) of the second group of biopolymerg compositions (hatched bars represent samples that remained unbroken during the test).

In the case of flexural tests (Figure 5 and Table S3), the usage of a mixture of ECE and HS (M12) did not affect σ_FT_ and ductility of the PLA matrix (M1) and only marginally increased its flexibility. The incorporation of mineral particles into such a matrix (M13–M15) reduced its flexibility, with the largest increase in E_F_ being observed for the systems containing talc (an increase by 500–1000 MPa, depending on the filler’s content)—these systems also exhibited the highest values of σ_F_ (i.e., the values increased by 12 MPa compared to the reference samples). Similar to tensile tests, the samples based on the blends of PLA with other polyesters, especially PBAT (M16, M17, M19), turned out to be the least resistant to the vertically applied external force. It is worth noting that the obtained data suggest that all systems containing either polyester blends or inorganic fillers were more ductile during bending, since they did not fracture up to the ε_F_ of 25%.

Charpy impact tests (Figure 4 and Table S4) evidenced that among the materials containing 0.75 wt % of ECE and 2 wt % of HS, the highest ability to impact energy dissipation was detected in those exhibiting the lowest values of *E*_T_ and *E*_F_ combined with the highest ductility during bending. In this area, the best performance was achieved by the system containing PLA and PBAT blends as well as talc particles (M17), for which the impact strength increased more than 10 times compared to the reference sample (M1 or M12). It is very important to underline that only these samples broke partially in the Charpy test. That outstanding result suggests that material M17 is predestined as the component of one of the bottle layers that improves impact resistance, especially since PLA has a very low resistance to dynamic stresses.

In general, one can say that the introduction of ECE into (filled) polyesters improves the mechanical strength of materials, whereas the additional application of HS slightly reduces it but improves ductility, elasticity, and impact properties. Based on mechanical tests and GPC analysis, it is possible that ECE quickly reacts with chain ends of polyesters and its hydroxyl groups originating from epoxide ring opening and forms a kind of three-dimensional scaffold or reinforcement of high molar mass in the material. HS seems to be less reactive than ECE at the stage of processing, so after that, the majority of HS remains unreacted in material. HS and its hydrated urea-like derivative act as a kind of plasticizer or lubricant (low molar mass fraction). It causes the decrease in composite strength due to the low molecular mass of HS and its expected immiscibility with the aliphatic polyester matrix (aromatic rings and unsaturated carbodiimide/urea moieties vs aliphatic chains with ester groups).^35^ On the other hand, it will ensure hydrolytic stability of the material in the long term.^36,37^ To demonstrate the effectiveness of the HS additive, accelerated hydrolysis tests were conducted for two compositions differing only in the addition of HS, namely compositions M9 and M13. The bar-shaped samples were immersed in water and incubated at 65 °C for 7 and 14 days. After this time, their mechanical strength was determined using a three-point bending test. Figure 6 (and Table S5) summarizes the results. After 7 days, a drastic decrease in the strength of composition M9 (42.9 MPa) was observed, corresponding to a loss of over 60% of the initial strength. At the same time, composition M13 retained significantly higher mechanical properties (102.7 MPa), exhibiting only a moderate decrease in strength. The difference between the tested materials becomes particularly pronounced at this stage. After 14 days of incubation, degradation of composition M9 is almost complete, with the flexural strength dropping to 0.3 MPa, indicating a practical loss of the material’s mechanical integrity. However, the M13 composition still retains significant strength (45.6 MPa), confirming the overall trend of the additive’s protective effect. In summary, the results clearly confirm that the presence of HS significantly slows down the hydrolytic degradation of the composite, enabling longer retention of its mechanical properties under accelerated aging conditions.

**Figure 6:**
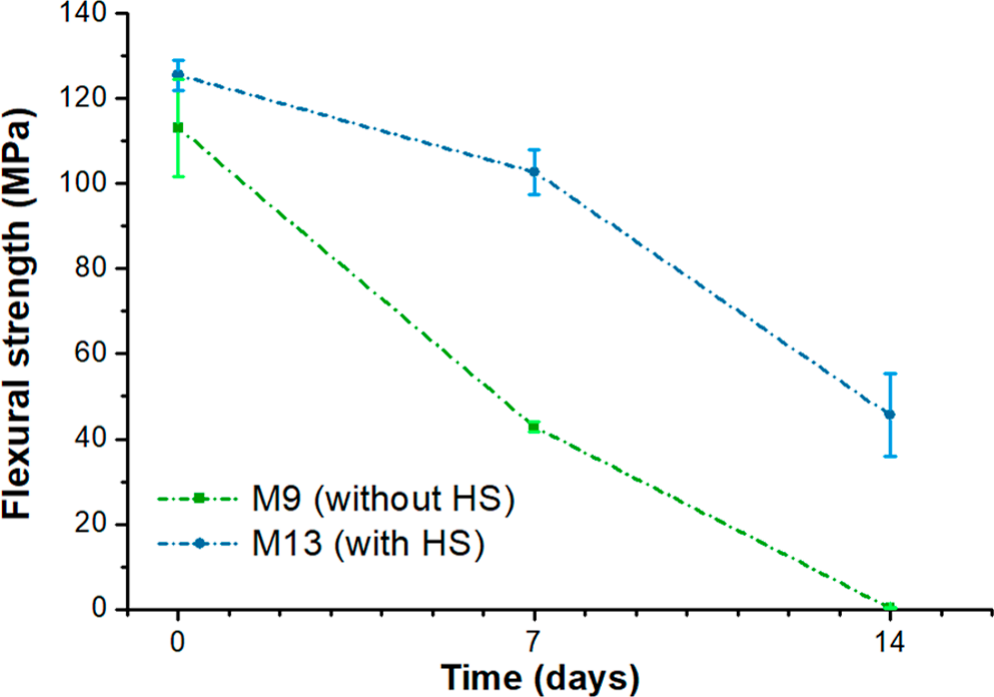
Flexural strength values for selected compositions after accelerated hydrolytic studies (water, 65 °C).

### Thermal Properties

To characterize the thermal properties of the materials, the glass transition of the amorphous phases of polymers (*T*_g_), the melting point (*T*_m_), the maximum crystallization ability (*X*_max_), and the postprocessing degree of crystallinity (*X*_c_) of the PLA were determined, as shown in Table S6 and Figure 7. The DSC profiles of the respective samples are presented in Figures S13–S31. Parameters regarding the degree of crystallinity: *X*_max_ and *X*_c_ were calculated using eqs 1 and ([Disp-formula eq2]), respectively

1 $$X_{\max}=\frac{{\Delta H}_{m}}{{\Delta H}_{m}^{ref}\times \omega_{PLA}}$$

2 $$X_{c}=\frac{{\Delta H}_{m}-{\Delta H}_{cc}}{{\Delta H}_{m}^{100\%}\times \omega_{PLA}}$$

where Δ*H*_cc_ is the enthalpy of cold crystallization of the PLA phase, Δ*H*_m_ and Δ*H*_m_^100%^ are the enthalpies of PLA α-phase melting of the sample studied and for totally α-crystalline L-PLA estimated as 93 J g^–1^,^38^ respectively, and ω_PLA_ is the weight fraction of PLA in the sample.

**Figure 7:**
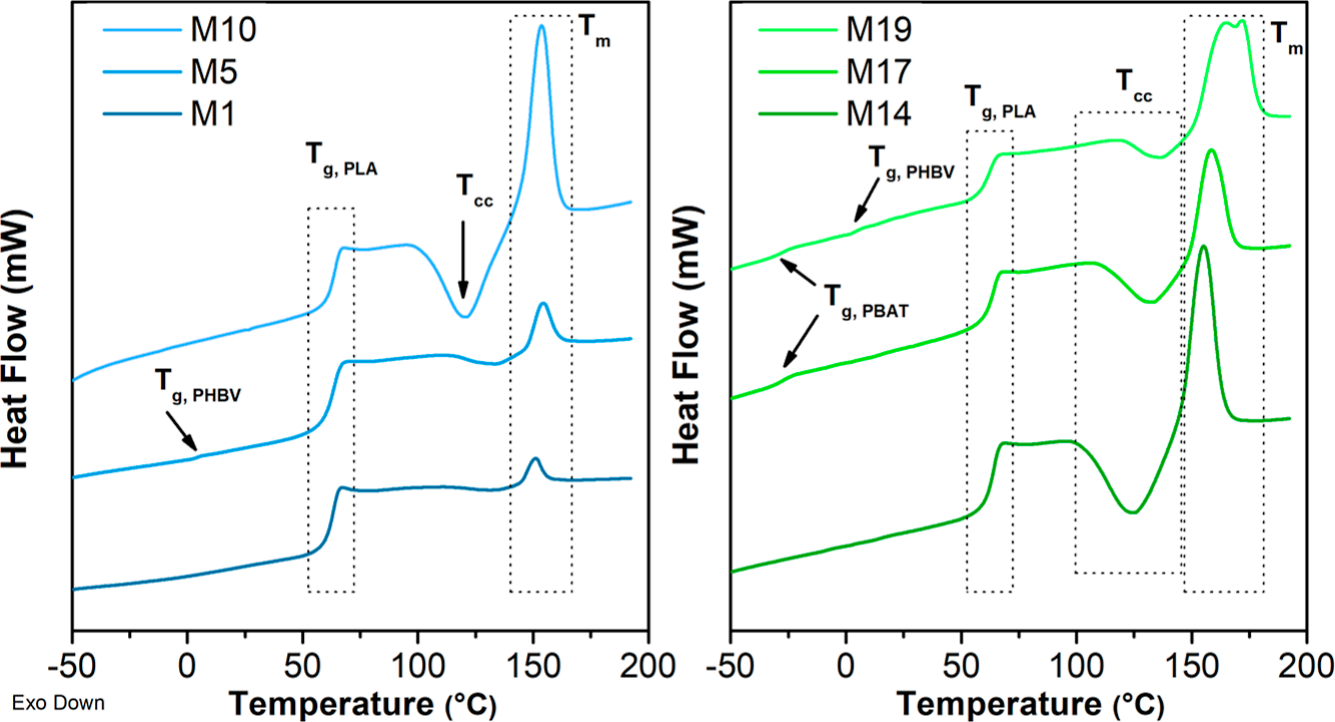
Graphical overview of selected samples obtained during DSC analysis.

For the first group of composites M1–M11, *T*_g_ values corresponding to individual polymer domains are observed, including PLA (*T*_g_ = 62–65 °C), PBAT (*T*_g_ = −30 °C) and PHBV (*T*_g_ = 3–6 °C). These values are equal (close) to the ones for pure components. This proves that the polymeric ingredients are immiscible with PLA, forming separate phases without or with very minor interphase. It is appreciated from the tensile strength and impact strength properties’ point of view. *T*_m_ values are within the range of 151–153 °C, in the case of pure PLA (M1) or samples without talc. It has been shown that the addition of ECE usually increases the degree of crystallinity of the systems (*X*_c_ increase), which is clearly seen in the example of compositions M2 and M3. A similar effect has already been found in literature.^39^ However, in the case of composites with talk (M10, M11), the temperature increases to 157 °C, which shows that talc is a good nucleating agent for the PLA α-phase, which assures a higher order of crystalline phase and a higher degree of crystallization (especially sample M11). The addition of chalk did not reveal such a phenomenon. Moreover, the addition of a filler such as talc (M10 and M11) also affects the increase in *X*_c_ compared to chalk (M7-M9).

All composites in the second group of composites (M12-M19) had an addition (2 wt %) of a HS. *T*_g_ values corresponding to individual polymer domains are observed, including PLA (*T*_g_ = 58–65 °C), PBAT (*T*_g_ = −42 to −28 °C) and PHBV (*T*_g_ = 3–6 °C). This proves that the mentioned components form partially immiscible polymer blends. It can be seen that the addition of a filler such as chalk reduces the *T*_g_ value coming from the PLA domains to below 60 °C, which can be noticed for the compositions M13 and M16. The *T*_m_ oscillates between 150 and 160 °C. Also, in this case, an increase in the value of this parameter is visible with the addition of talc used as a filler. No clear trends in crystallinity parameters resulting from differences in the compositions of the produced (bio)­composites were identified.

Thermal stability of these compositions was studied by means of TGA under an inert atmosphere but without postprocessing sample drying. The temperatures of weight loss of 5 and 10 wt % of the samples (*T*_5%_, *T*_10%_) and maximum rate(s) of decomposition (*T*_max_), as well as wt % of residue, were determined (Table S7, Figure 8). The TGA profiles of the respective samples are presented in Figures S13–S31.

**Figure 8:**
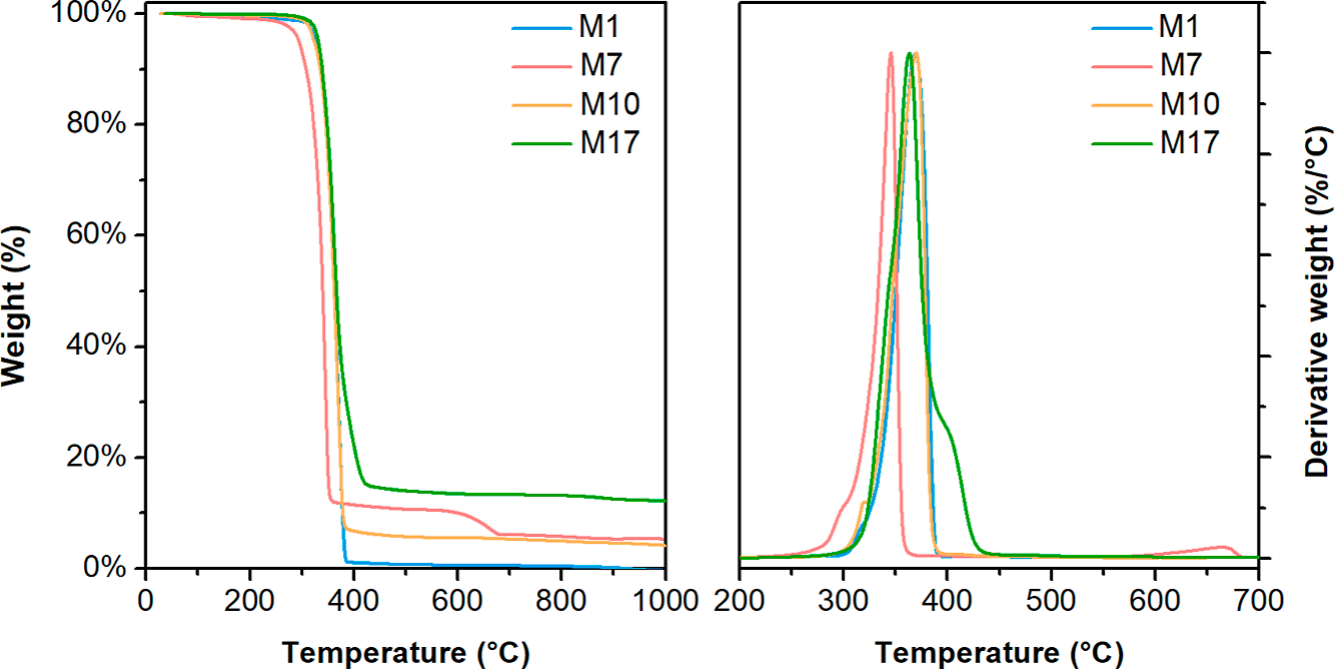
Graphical overview of TGA thermograms (left panel) and DTG (right panel, narrow temperature range with effects visible) of selected samples.

The 5% and 10% loss temperatures depend mainly on material composition. In the case of *T*_5%_, the range is from 250 to 336 °C, while for *T*_10%_, it is from 269 to 342 °C. The lowest temperature value at 5% mass loss is characteristic of samples that contain only one type of biopolymer, i.e., PLA, and the addition of CaCO_3_ filler. This phenomenon may be caused by the catalytic properties of the hygroscopic filler, which may accelerate thermal degradation of the polymer via hydrolysis, transesterification, and cyclization-depolymerization.^40,41^ The maximum decomposition temperatures also depend directly on the composition of the (bio)­composition. In the first stage of degradation (200–400 °C), the organic phase of the materials is decomposed in the order from the least thermally stable PHBV (*T*_max_ = 256 °C), then the decomposition of PLA (*T*_max_ = 335 °C), and finally the aromatic part of PBAT (*T*_max_ = 407 °C).^35,36^ In subsequent stages of decomposition, aromatic groups contained in functional additives such as ECE or HS may degrade. The final stage, taking place at higher temperatures (*T*_max_ = 743 °C), is the decomposition of inorganic fillers such as chalk (decomposition to CaO with the release of CO_2_), while talc does not decompose under the measurement conditions. For these reasons, compositions using inorganic fillers show little residual mass. Unexpectedly, the addition of the HS slightly reduces *T*_5%_, *T*_10%_, and *T*_max_ compared to the materials without HS, which may be a result of volatile emission associated with the degradation of HS itself (Figure S32). Literature shows that HS should improve thermal stability under processing conditions,^37^ but in higher temperatures, the benefits appear to be limited. Moreover, as can be expected, the addition of polymers with lower thermal stability (PHBV) negatively affects the thermal stability of the blend compared to pure PLA. However, a positive effect on the thermal stability of materials with the addition of talc is visible, in contrast to CaCO_3_. Also, the presence of ECE reveals a stabilization effect. Besides the differences in thermal stability and the effects of the various components, all the samples studied exhibited sufficiently high temperatures—*T*_5%_ (>250 °C), *T*_10%_ (>268 °C), and *T*_max1_ (>272 °C)—to allow efficient material processing and forming.

### Rheological Properties

The basic way to determine the processing properties of polymer materials is to determine their melt flow rates (MFR). Knowledge of these rates is crucial in selecting the appropriate processing parameters, including the temperature of individual zones or pressure in the barrel. In the EBM technique, melt resistance and shape stability during blow molding are extremely important. Low MFR materials have a higher viscosity, which helps maintain uniform wall thickness and avoid deformation and parison rupture. Based on our experience with manufacturing HDPE bottles via EBM, we assumed the optimum MFI range as 0.1–3 g/10 min, depending on the type of plastic and the requirements of the final product. Figure 9 and Table S8 present a summary of the MFR values for all obtained composites. As expected, the introduction of ECE to pure PLA (M1) significantly reduces the MFR index (M2, M3) and enables us to use PLA for EBM. It has been shown that the introduction of PBAT and PHBV to the mixtures causes a significant increase in the MFR value. In the case of PBAT, which reveals similar MFR to PLA, the key role may be played by immiscibility of components; therefore, dispersed drops of molten PBAT act as interfacial slip for PLA, reducing shear friction. PHBV is characterized by MFR (8–15 g/10 min), higher than that for PLA, and moreover, based on DSC results (separate glass transitions for PLA and PHBV), it might also be immiscible with PLA in melt. Similarly, incorporation of CaCO_3_ filler causes a drastic increase in the MFR value compared to the M1 (pure PLA). That phenomenon may be attributed to the “lubrication slipping” effect of finely dispersed CaCO_3_ particles, which reduce intermolecular and interparticle shear friction within the melt, thereby facilitating flow.^42^ The addition of ECE compensates for viscosity loss both in the case of adding polyesters and chalk. The systems comprising ECE along with HS reveal similar trends with respect to their compositions; however, generally, they exhibit MFR values appropriate for EBM. The elevated values are observed for two compositions containing chalk.

**Figure 9:**
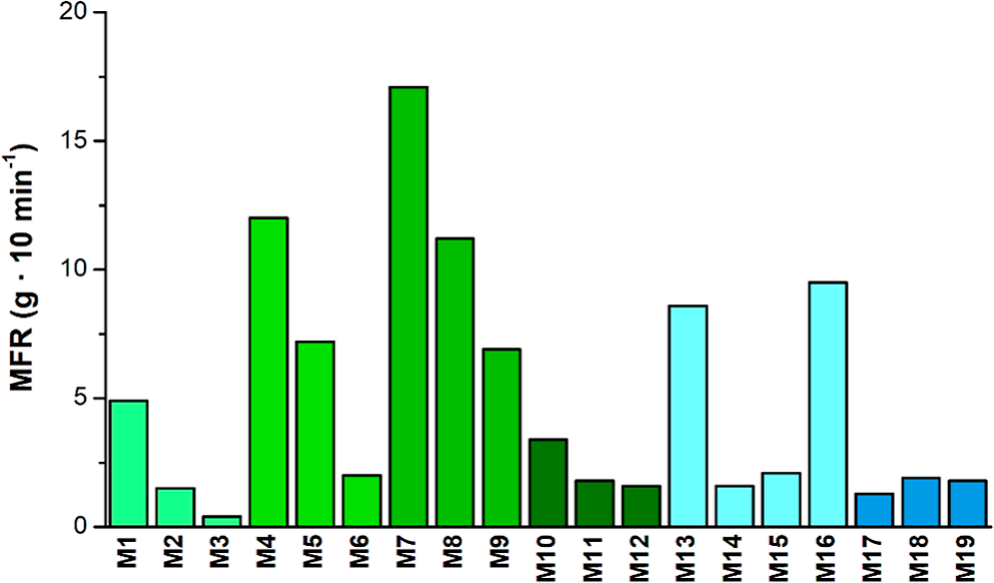
Rheological properties (MFR value) of biopolymer compositions.

### Morphology Analysis

Figure 10 and figures in Table S9 present the images of the fracture surfaces of the studied materials, observed via scanning electron microscopy. The sample of neat PLA (M1) shows a brittle, smooth fracture, while the introduction of ECE (M2, M3) brings more fibrous, cohesive ones. The indications of that microfibril-like structure are seen in all samples which contain ECE, due to the formation of long and branched polymer chains, capable of higher deformation before fracture. Introduction of PBAT elastomer to the material (M4 and in multicomponent systems M16–M17–M19) exhibits spherical dispersed-phase domains that often undergo cavitation upon fracture, accompanied by shear-yield bands in the matrix, with the relatively high interfacial tension between PLA and PBAT making these domains well distinguishable in SEM images. On the other hand, PHBV (M5, M6, M18) forms smaller, less contrasting domains whose boundaries often blend with the PLA matrix in SEM, making them harder to distinguish compared to PBAT. Comparing the two applied fillers, CaCO_3_ (M7–M9; with HS/ECE also M13, M16, M18) shows submicron- to micron-sized particles with pull-outs and voids where adhesion to the matrix is weaker, while ECE/HS reduces such voids and increases cohesive fracture at the particle interface, whereas talc (M10–M11; and M14–M15, M17, M19) forms plate-like lamellae that often cause crack deflection, producing rougher fracture surfaces and visible platelet orientation even on original surfaces. Discussing HS performance (M12–M19, always with 0.75% ECE), it generally forms a more “cohesive” morphology with fewer particle pull-out voids (especially with CaCO_3_) and more cohesive fractures. In PBAT-containing systems, the domains tend to be smaller and more firmly anchored in the matrix (synergy of HS with ECE), which visually reduces the sharpness of their boundaries.

**Figure 10:**
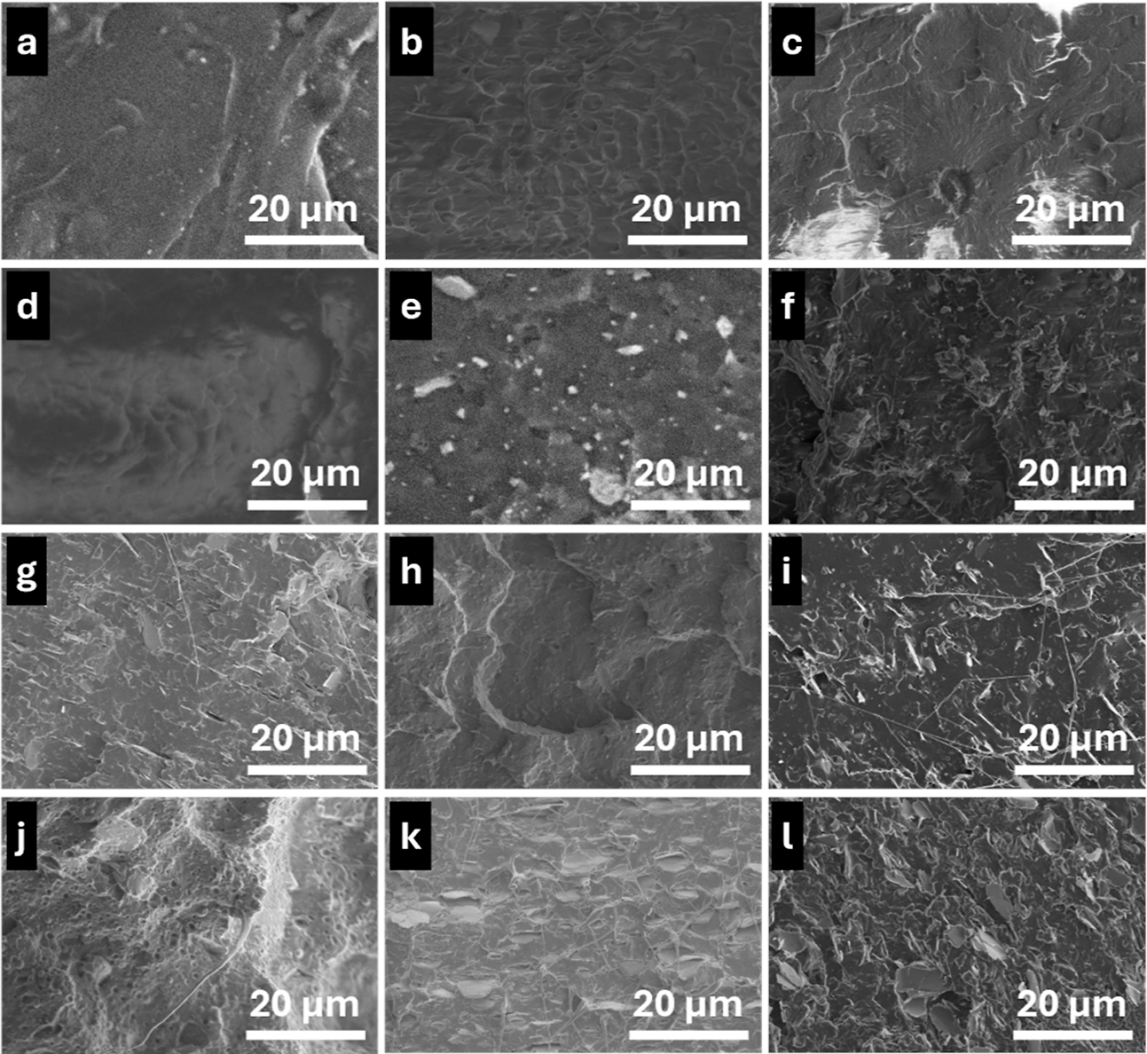
SEM images of the fracture surfaces of compositions: (a) M1, (b) M3, (c) M5, (d) M6, (e) M7, (f) M8, (g) M11, (h) M13, (i) M14, (j) M16, (k) M18, and (l) M19.

## Conclusions

This study demonstrates that an epoxy-based chain extender (ECE) is the most effective route to enhance molar mass and branching in polyesters, including PLA, PHBV, and PBAT, enabling mechanical reinforcement and stable processing via extrusion blow molding. Carbodiimide-based HS, while broadening dispersity and slightly reducing average molar masses, acts in part as a lubricant and provides a latent capacity for long-term hydrolytic stabilization rather than direct mechanical improvement. Among mineral fillers, talc supports higher Mw and favorable fracture morphologies through crack deflection, whereas CaCO_3_ tends to catalyze molar mass reduction; therefore, talc appears to be the best choice in the studied systems. PBAT enhances ductility and impact strength through cavitation-driven plasticity, while PHBV contributes finer, less distinct domains with more subtle effects on toughness. All applied polyesters exhibit mutual immiscibility in DSC, implying that PBAT and PHBV function primarily as impact strength modifiers rather than plasticizers. Overall, the most promising formulations for compostable bottle-layer applications combine PLA with ECE, optional HS for durability, and talc as a reinforcing filler, with PBAT or PHBV additions fine-tuning the toughness–strength–processability balance.

On the basis of the results obtained, specific mixtures were selected for future application in the production of a multilayer bottle. The good candidate for the outer barrier, providing protection against contact with the user and the external environment, would be the M10 composition, which is characterized by good mechanical performance and a relatively simple formulation. The middle layer, ensuring the required mechanical strength and high impact strength, could be represented by the M17 or M19 samples, which exhibit enhanced fracture toughness due to the incorporation of 20 wt % PBAT or 30 wt % mixed PBAT/PHBV. The inner barrier, designed to resist exposure to various chemical agents, could be the M14 composition, composed of ECE and talc-reinforced PLA containing 2 wt % HS, which helps mitigate the adverse effects of household chemicals on the final product.

## Supplementary Material



## Notes

Summary of molar mass parameters obtained using GPC analysis; mathematical treatment involving numerical fitting of two or three Gaussian subdistributions, forming approximately the original curve, for selected samples; molar mass distribution for HS and HS urea-like; FTIR spectra of HS and its partial urea-like derivative, M2 without HS and M12 containing HS; strength parameters obtained in the tensile test, three-point bending test, and impact test; flexural strength values for selected compositions after accelerated hydrolytic studies; summary of parameters obtained by DSC and TGA analysis for materials; thermal analysis of samples: DSC curves and TGA curves; thermogravimetric analysis of HS; summary of the MFR results of obtained polymer mixtures; and SEM images of compositions M1–M19 (as-manufactured surfaces and fractures) (PDF)
The data, including raw GPC analysis reports and an Excel spreadsheet with statistical tests of the strength data, are available in the “WUT Research Data Repository.’ File link: DOI: 10.71724/maj4-rh44

## References

[1] British Plastics Federation Plastics Applications; British Plastics Federation. https://www.bpf.co.uk/plastipedia/applications/Default.aspx (accessed Feb 05, 2024).

[2] Andrady A.
L., Neal M. A. (2009). Applications
and Societal Benefits of Plastics. Philos. Trans.
R. Soc., B.

[3] Sundqvist-Andberg H., Åkerman M. (2021). Sustainability Governance and Contested Plastic Food
Packaging – An Integrative Review. J.
Cleaner Prod..

[4] Auras, R. A. ; Lim, L.-T. ; Selke, S. E. M. ; Tsuji, H. Poly(Lactic Acid): Synthesis, Structures, Properties, Processing, and Applications; John Wiley & Sons, 2010.

[5] Gironi F., Piemonte V. (2011). Bioplastics and Petroleum-Based Plastics:
Strengths
and Weaknesses. Energy Sources, Part A.

[6] Vink E. T. H., Rábago K. R., Glassner D. A., Gruber P. R. (2003). Applications
of Life Cycle Assessment to NatureWorks^TM^ Polylactide (PLA)
Production. Polym. Degrad. Stab..

[7] Tokiwa Y., Calabia B. P., Ugwu C. U., Aiba S. (2009). Biodegradability of
Plastics. Int. J. Mol. Sci..

[8] OECD . Global Plastics Outlook: Economic Drivers, Environmental Impacts and Policy Options; OECD, 2022.

[9] Wu W.-M., Yang J., Criddle C. S. (2017). Microplastics
Pollution and Reduction
Strategies. Front. Environ. Sci. Eng..

[10] Agarwal S. (2020). Biodegradable
Polymers: Present Opportunities and Challenges in Providing a Microplastic-Free
Environment. Macromol. Chem. Phys..

[11] Plastics Europe . Plastics—The Fast Facts 2023, 2024. https://plasticseurope.org/knowledge-hub/plastics-the-fast-facts-2023/ (accessed Feb 05, 2024).

[12] EUBIO_Admin . Market; 2024. https://www.european-bioplastics.org/market/ (accessed Feb 21, 2024).

[13] European Commission . Plastics—European Commission. https://environment.ec.europa.eu/topics/plastics_en (accessed Feb 05, 2024).

[14] European Commission . European Green Deal: Putting an end to wasteful packaging. https://ec.europa.eu/commission/presscorner/detail/en/ip_22_7155 (accessed Feb 05, 2024).

[15] US EPA Plastics: Material-Specific Data; Collections and Lists, 2017. https://www.epa.gov/facts-and-figures-about-materials-waste-and-recycling/plastics-material-specific-data (accessed Feb 07, 2024).

[16] Atiwesh G., Mikhael A., Parrish C. C., Banoub J., Le T.-A. T. (2021). Environmental
Impact of Bioplastic Use: A Review. Heliyon.

[17] Renewable Carbon News Poster Update Biodegradable Polymers in Various Environments According to Established Standards and Certification Schemes; Renewable Carbon News, 2021.

[18] Mo A., Zhang Y., Gao W., Jiang J., He D. (2023). Environmental
Fate and Impacts of Biodegradable Plastics in Agricultural Soil Ecosystems. Appl. Soil Ecol..

[19] Jian J., Xiangbin Z., Xianbo H. (2020). An Overview
on Synthesis, Properties
and Applications of Poly­(Butylene-Adipate-*Co*-Terephthalate)–PBAT. Adv. Ind. Eng. Polym. Res..

[20] Cheng J., Eyheraguibel B., Jacquin J., Pujo-Pay M., Conan P., Barbe V., Hoypierres J., Deligey G., Halle A. T., Bruzaud S., Ghiglione J.-F., Meistertzheim A.-L. (2022). Biodegradability
under Marine Conditions of Bio-Based and Petroleum-Based Polymers
as Substitutes of Conventional Microparticles. Polym. Degrad. Stab..

[21] Benavides P. T., Lee U., Zarè-Mehrjerdi O. (2020). Life Cycle
Greenhouse Gas Emissions
and Energy Use of Polylactic Acid, Bio-Derived Polyethylene, and Fossil-Derived
Polyethylene. J. Cleaner Prod..

[22] Guilbert, S. ; Guillaume, C. ; Gontard, N. New Packaging Materials Based on Renewable Resources: Properties, Applications, and Prospects. In Food Engineering Interfaces; Aguilera, J. M. , Simpson, R. , Welti-Chanes, J. , Bermudez-Aguirre, D. , Barbosa-Canovas, G. , Eds.; Springer: New York, NY, 2011; pp 619–630.

[23] Walker S., Rothman R. (2020). Life Cycle Assessment
of Bio-Based and Fossil-Based
Plastic: A Review. J. Cleaner Prod..

[24] Auras R. A., Singh S. P., Singh J. J. (2005). Evaluation of Oriented
Poly­(Lactide)
Polymers vs. Existing PET and Oriented PS for Fresh Food Service Containers. Packag. Technol. Sci..

[25] Auras R., Harte B., Selke S. (2004). An Overview of Polylactides as Packaging
Materials. Macromol. Biosci..

[26] Ishimwe, S. European Bioplastics collaborates with nova-Institute and Plastics Europe to deliver bioplastics production data for 2022; European Bioplastics e.V. https://www.european-bioplastics.org/european-bioplastics-collaborates-with-nova-institute-and-plastics-europe-to-deliver-bioplastics-production-data-for-2022/ (accessed Feb 07, 2024).

[27] Chamas A., Moon H., Zheng J., Qiu Y., Tabassum T., Jang J. H., Abu-Omar M., Scott S. L., Suh S. (2020). Degradation
Rates of Plastics in the Environment. ACS Sustain.
Chem. Eng..

[28] de
Jong S. J., Arias E. R., Rijkers D. T. S., van
Nostrum C. F., Kettenes-van den Bosch J. J., Hennink W. E. (2001). New Insights
into the Hydrolytic Degradation of Poly­(Lactic Acid): Participation
of the Alcohol Terminus. Polymer.

[29] Jamshidian M., Tehrany E. A., Imran M., Jacquot M., Desobry S. (2010). Poly-Lactic
Acid: Production, Applications, Nanocomposites, and Release Studies. Compr. Rev. Food Sci. Food Saf..

[30] Barletta M., Aversa C., Puopolo M., Vesco S. (2019). Extrusion Blow Molding
of Environmentally Friendly Bottles in Biodegradable Polyesters Blends. Polym. Test..

[31] Bikiaris D. N., Triantafyllidis K. S. (2013). HDPE/Cu-Nanofiber
Nanocomposites with Enhanced Antibacterial
and Oxygen Barrier Properties Appropriate for Food Packaging Applications. Mater. Lett..

[32] Hong S.-I., Krochta J. M. (2006). Oxygen Barrier Performance
of Whey-Protein-Coated Plastic
Films as Affected by Temperature, Relative Humidity, Base Film and
Protein Type. J. Food Eng..

[33] Florjańczyk Z., Jóźwiak A., Kundys A., Plichta A., Dębowski M., Rokicki G., Parzuchowski P., Lisowska P., Zychewicz A. (2012). Segmental
Copolymers of Condensation
Polyesters and Polylactide. Polym. Degrad. Stab..

[34] Li L., Wang H., Jiang T., Hu C., Zhang J., Zheng H., Zeng G. (2023). Effect of Epoxy Chain
Extenders on
Molecular Structure and Properties of Polylactic Acid. J. Appl. Polym. Sci..

[35] Zhang Q., Cai H., Zhang A., Lin X., Yi W., Zhang J. (2018). Effects of
Lubricant and Toughening Agent on the Fluidity and Toughness of Poplar
Powder-Reinforced Polylactic Acid 3D Printing Materials. Polymers.

[36] Porfyris A., Vasilakos S., Zotiadis C., Papaspyrides C., Moser K., Van der
Schueren L., Buyle G., Pavlidou S., Vouyiouka S. (2018). Accelerated
Ageing and Hydrolytic Stabilization of
Poly­(Lactic Acid) (PLA) under Humidity and Temperature Conditioning. Polym. Test..

[37] Yang L., Chen X., Jing X. (2008). Stabilization
of Poly­(Lactic Acid)
by Polycarbodiimide. Polym. Degrad. Stab..

[38] Turner J. F., Riga A., O’Connor A., Zhang J., Collis J. (2004). Characterization
of Drawn and Undrawn Poly-L-Lactide Films by Differential Scanning
Calorimetry. J. Therm. Anal. Calorim..

[39] Arruda L. C., Magaton M., Bretas R. E. S., Ueki M. M. (2015). Influence of Chain
Extender on Mechanical, Thermal and Morphological Properties of Blown
Films of PLA/PBAT Blends. Polym. Test..

[40] Jia X., Wen Q., Sun Y., Chen Y., Gao D., Ru Y., Chen N. (2024). Preparation
and Performance of PBAT/PLA/CaCO3 Composites via Solid-State
Shear Milling Technology. Polymers.

[41] Kim H.-S., Park B. H., Choi J. H., Yoon J.-S. (2008). Mechanical Properties
and Thermal Stability of Poly­(L-Lactide)/Calcium Carbonate Composites. J. Appl. Polym. Sci..

[42] Nekhamanurak B., Patanathabutr P., Hongsriphan N. (2014). The Influence of Micro-/Nano-CaCO3
on Thermal Stability and Melt Rheology Behavior of Poly­(Lactic Acid). Energy Procedia.

