# Supplementary material for: Toward Compostable Packaging: Biodegradable Polymer Blends with Biobased Components for Household Chemical Bottle-Layer Applications
Source: ACS Omega. 2026 Jul 7;11(28):41182–94. doi: 10.1021/acsomega.5c10689 (PMC13393179; doi:10.1021/acsomega.5c10689)
Supplement: Supplementary file 1 [file ao5c10689_si_001.pdf]

Supporting Information

Towards compostable packaging: biodegradable polymer blends with bio-based components for household chemical bottle-layer applications

Sebastian Kowalczyk [a], Matylda Szewczyk-Łagodzińska [a], Maciej Dębowski [a], Anna Iuliano [a], Monika Truskolaska [b], Maria Krywult-Pawlik [b], Gabriela Jędrzejczak [b], Natalia Grochowska [a], Andrzej Plichta [a]*

[a] Warsaw University of Technology, Faculty of Chemistry, Chair of Polymer Chemistry and Technology, Noakowskiego 3, 00-664 Warsaw, Poland

[b] GRUPA INCO S.A., Wspólna 25, 00-519 Warsaw, Poland

*Email: andrzej.plichta@pw.edu.pl

Table S1. Summary of molar mass parameters obtained using GPC analysis.

Material	Molar mass parameters for the entire distribution				Fitting peaks
	M_n (kg·mol^{-1})	M_w (kg·mol^{-1})	$Đ$	M_p (kg·mol^{-1})	M_p^{fit} (kg·mol^{-1})
neat PLA	87.4	199	2.27	162	-
neat PBAT	41.7	100	2.41	86.0	-
neat PHBV	103	245	2.39	218	-
neat HS	5.12	12.7	2.48	13.6	-
M1	83.5	196	2.35	149	-
M2	96.6	223	2.31	151	162^c<br>524^d
M3	120	352	2.93	245	253^c<br>910^d
M4	90.6	185	2.04	132	-
M5	77.4	169	2.18	91.5	79.0^c<br>144^d
M6	109	292	2.68	123	129^c<br>410^d<br>964^e
M7	52.0	153	2.94	106	-
M8	82.8	243	2.93	148	-
M9	61.1	225	3.68	171	-
M10	79.9	239	2.99	136	-
M11	61.8	229	3.71	148	-
M12	39.5	206	5.22	12.4^a<br>114^b	15.7^c<br>100^d<br>221^e
M13	22.3	114	5.11	102	-
M14	13.9	212	15.3	198	11.7^c<br>117^d
M15	50.9	216	4.24	12.1^a<br>179^b	18.7^c<br>230^d
M16	24.0	131	5.46	90.5	50.0^c<br>180^d<br>434^e
M17	13.9	190	13.7	175	-
M18	71.3	231	3.24	219	28.4^c<br>90.0^d<br>244^e
M19	17.1	182	10.6	157	39.3^c<br>207^d

Note: for M_p in case of samples characterized by distinguished peaks (more than one local maximum) of molar mass distributions, these peak values for ^{a} first and ^{b} second molar mass distribution are given separately; for multimodal samples with separate distribution fitted numerically the M_p^{fit} values for ^{c} first, ^{d} second and ^{e} third fitted molar mass distribution are given separately.

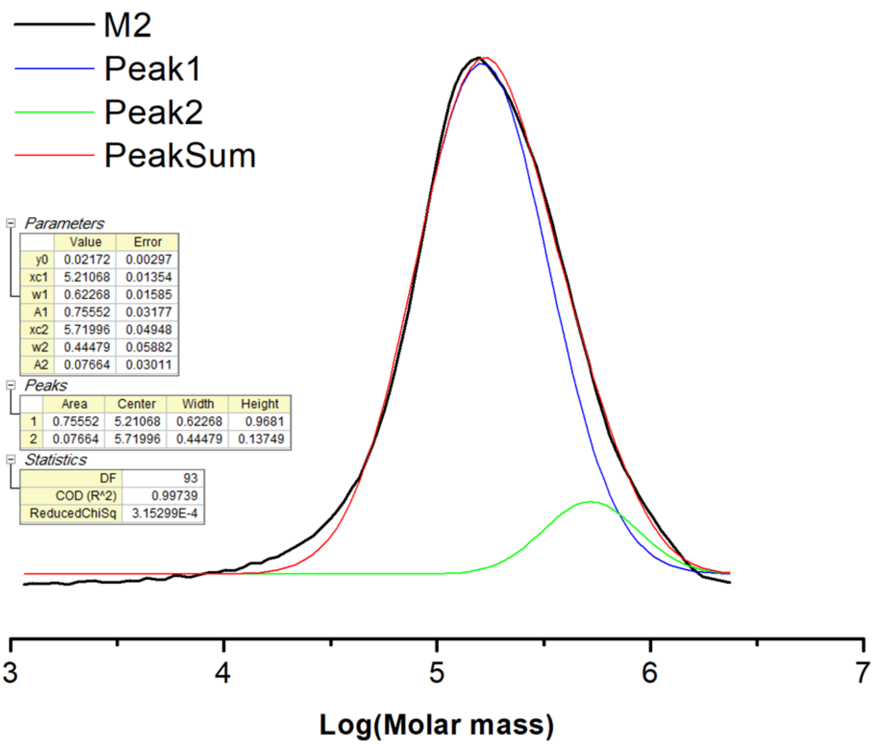

Figure S1. Mathematical treatment involving numerical fitting of two Gaussian subdistributions, forming approximately the original curve, for sample M2.

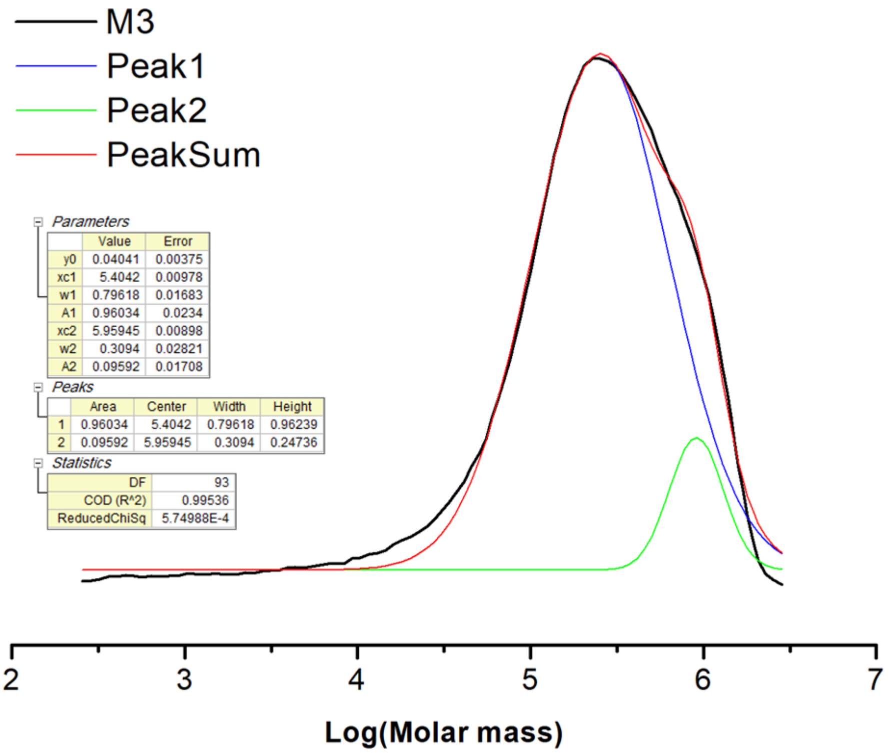

Figure S2. Mathematical treatment involving numerical fitting of two Gaussian subdistributions, forming approximately the original curve, for sample M3.

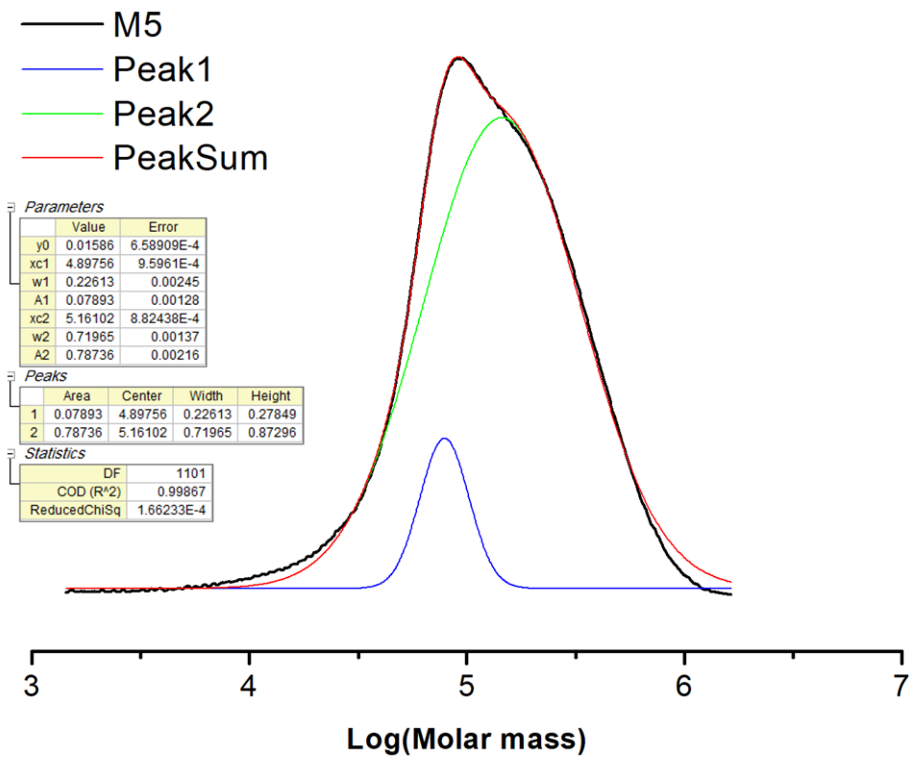

Figure S3. Mathematical treatment involving numerical fitting of two Gaussian subdistributions, forming approximately the original curve, for sample M5.

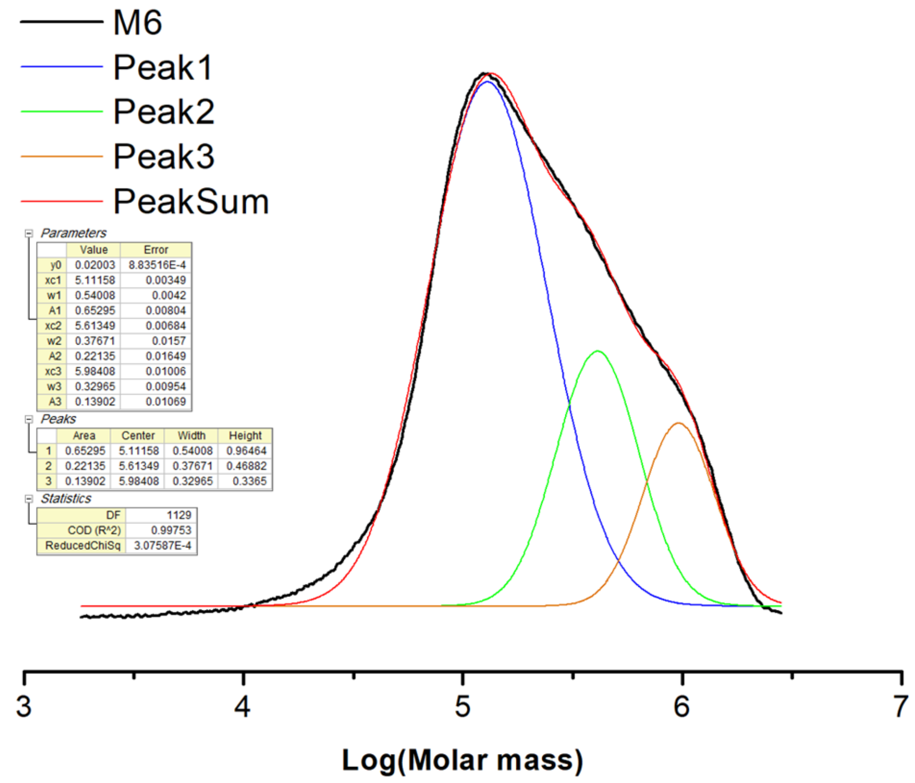

Figure S4. Mathematical treatment involving numerical fitting of three Gaussian subdistributions, forming approximately the original curve, for sample M6.

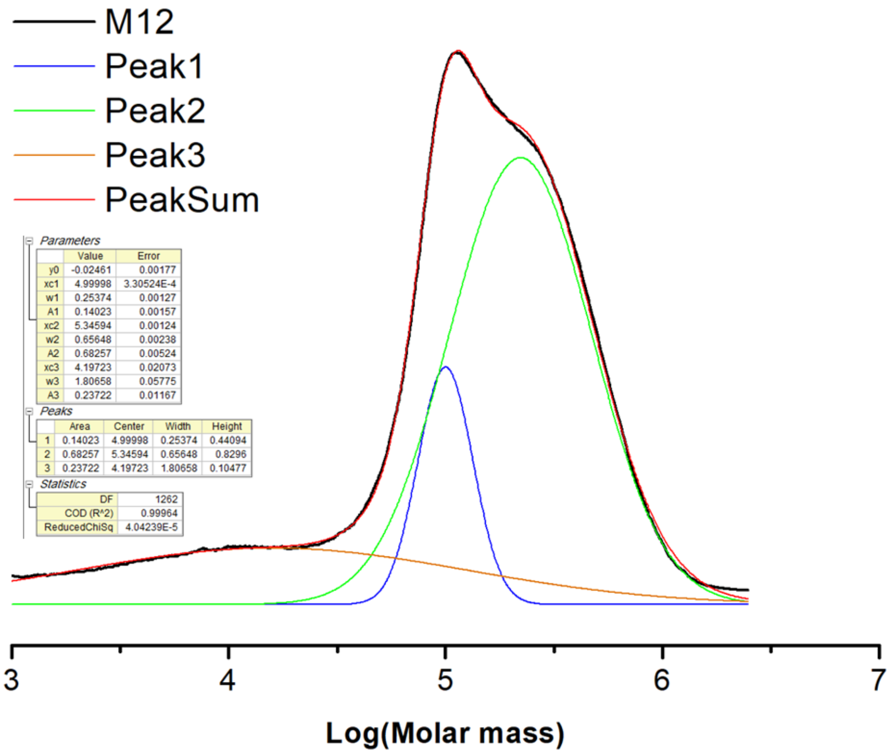

Figure S5. Mathematical treatment involving numerical fitting of three Gaussian subdistributions, forming approximately the original curve, for sample M12.

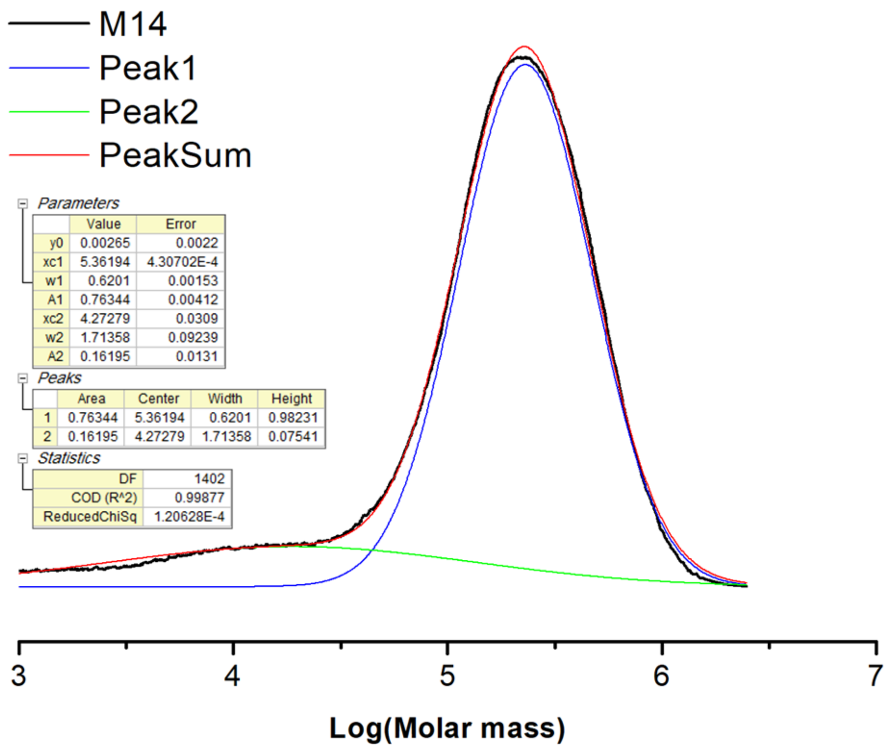

Figure S6. Mathematical treatment involving numerical fitting of two Gaussian subdistributions, forming approximately the original curve, for sample M14.

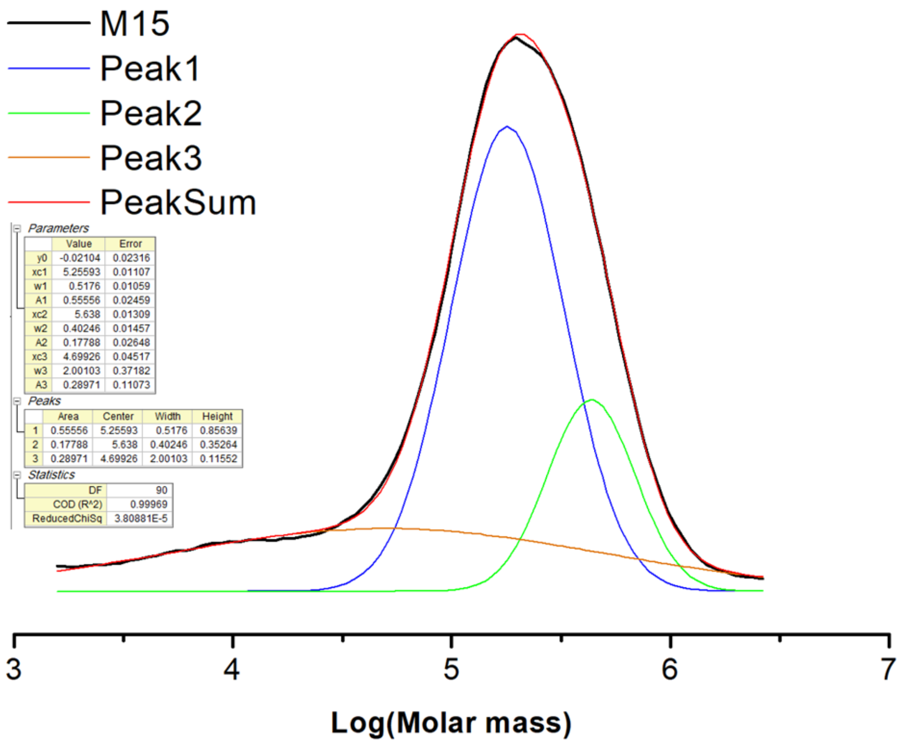

Figure S7. Mathematical treatment involving numerical fitting of three Gaussian subdistributions, forming approximately the original curve, for sample M15.

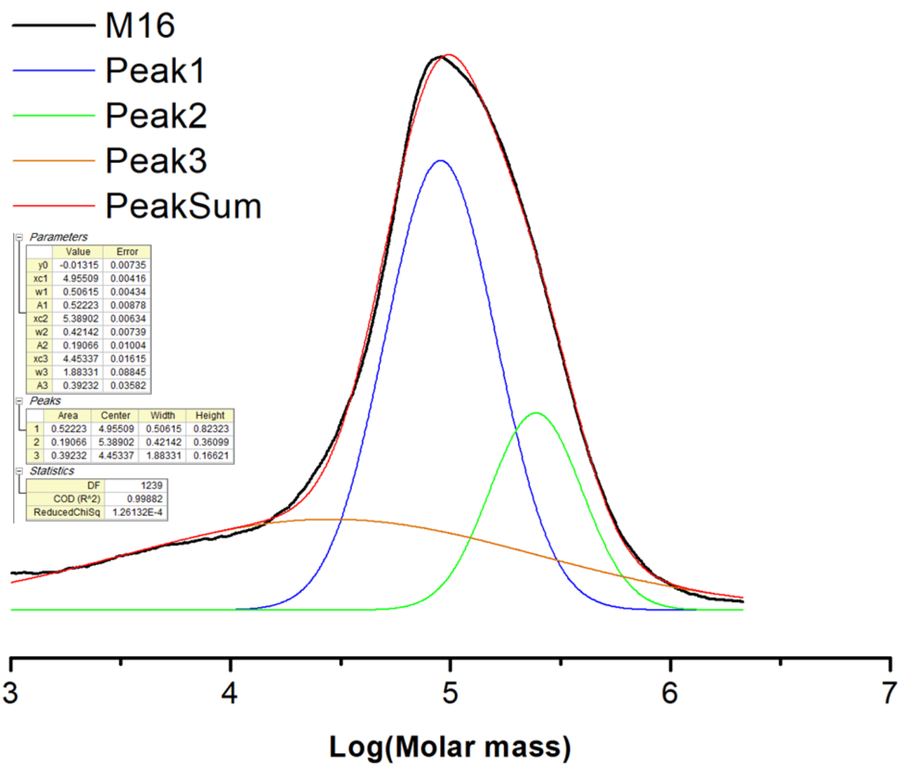

Figure S8. Mathematical treatment involving numerical fitting of three Gaussian subdistributions, forming approximately the original curve, for sample M16.

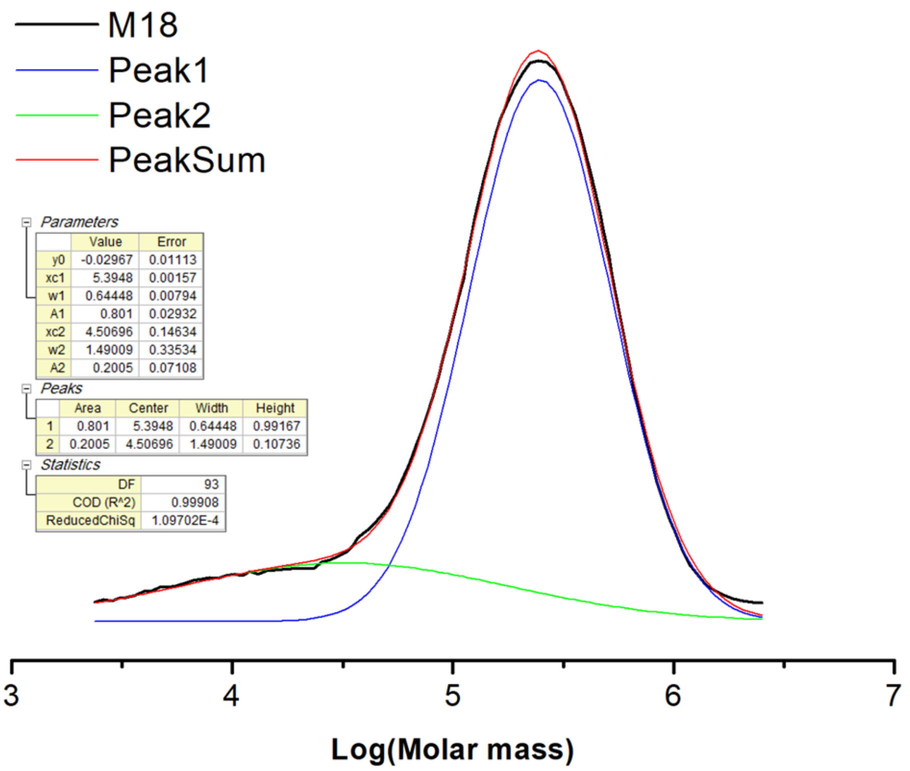

Figure S9. Mathematical treatment involving numerical fitting of two Gaussian subdistributions, forming approximately the original curve, for sample M18.

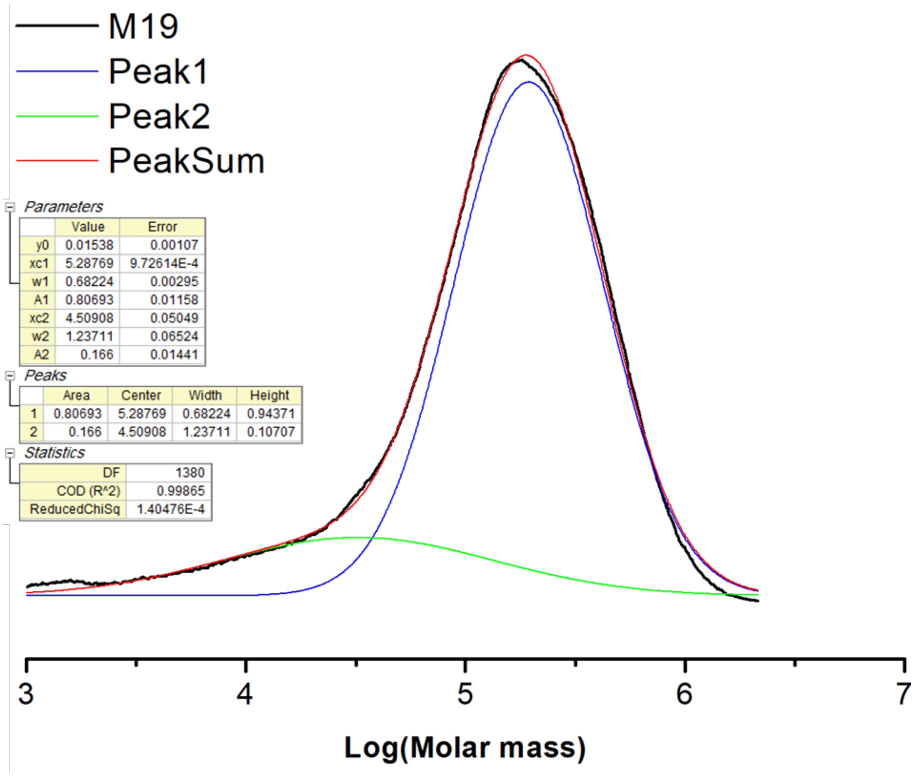

Figure S10. Mathematical treatment involving numerical fitting of two Gaussian subdistributions, forming approximately the original curve, for sample M19.

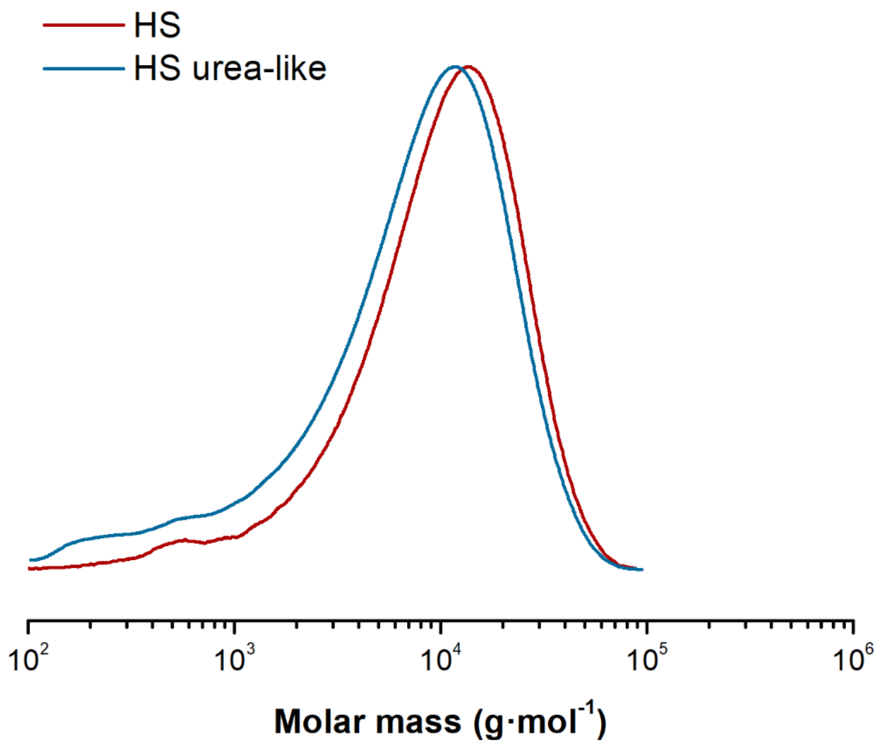

Figure S11. Molar mass distribution for HS and HS urea-like.

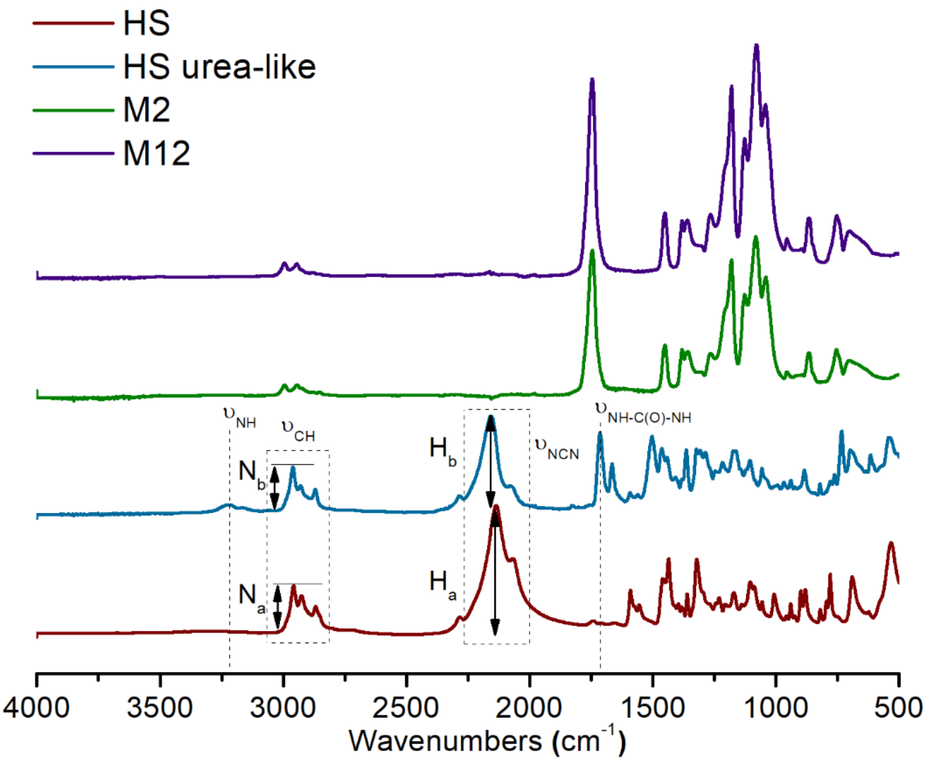

Figure S12. The FTIR spectra of HS and its partial urea-like derivative, M2 without HS and M12 containing HS ($N_b \cdot N_a^{-1} = 1$; $H_b \cdot H_a^{-1} = 0{,}77$).

Table S2. Strength parameters were obtained in the tensile test.

Material	Mechanical properties - Tensile strength					
	Tensile strength (MPa)		Elongation at break (%)		Young modulus (MPa)	
	Average	SD[a]	Average	SD[a]	Average	SD[a]
M1	68.7	1.6	14.0	2.2	721	36
M2	71.7	1.7	13.1	1.5	655	71
M3	73.9	3.0	12.2	2.6	717	55
M4	63.0	1.7	60.8	17	610	19
M5	64.6	1.4	30.6	23	667	80
M6	66.5	1.7	37.1	25	656	37
M7	60.0	1.2	15.8	4.8	745	23
M8	61.9	1.7	13.7	3.1	728	94
M9	65.4	2.0	11.1	2.6	779	50
M10	69.7	3.5	15.9	5.4	659	99
M11	70.5	2.8	19.3	4.6	683	79
M12	73.0	1.4	12.3	1.0	684	60
M13	59.2	1.6	13.4	4.0	723	66
M14	67.1	2.0	14.8	5.3	652	63
M15	67.2	1.0	16.9	4.7	694	48
M16	52.8	1.8	15.5	7.9	594	58
M17	50.5	0.9	95.9	25	473	21
M18	61.4	1.1	17.2	13	626	21
M19	50.8	2.3	124	62	497	35

Note: [a] SD – standard deviation

Table S3. Strength parameters were obtained in the three-point bending test.

Material	Mechanical properties - Flexural strength					
	Flexural strength (MPa)		Deflection at fracture (%)		Flexural modulus (MPa)	
	Average	SD[a]	Average	SD[a]	Average	SD[a]
M1	121.2	10.0	9.5	1.7	3652	27
M2	129.2	4.7	8.2	1.2	3654	102
M3	128.6	9.1	10.5	1.4	3566	113
M4	124.6	6.3	-[b]		3588	100
M5	115.4	4.9	-[b]		3367	59
M6	119.1	6.0	-[b]		3521	124
M7	106.9	11.2	17.6	4.1	4012	49
M8	121.9	6.0	13.7	4.4	4110	129
M9	113.1	11.4	8.3	0.9	3929	90
M10	117.6	11.8	-[b]		3979	77
M11	135.4	5.4	-[b]		5038	80
M12	125.1	8.3	9.5	5.3	3454	89
M13	125.4	3.6	21.2	5.4	3760	122
M14	130.5	4.3	18.7	6.5	4001	119
M15	137.4	2.9	-[b]		4786	141
M16	105.6	2.4	-[b]		3201	98
M17	100.9	1.5	-[b]		3202	72
M18	117.2	2.0	-[b]		3994	88
M19	105.6	3.8	-[b]		3285	80

Note: [a] SD – standard deviation; [b] indicates that the specimen did not undergo complete fracture (separation into two pieces) up to 25% strain; the test was terminated at this strain level.

Table S4. Impact strength results and type of destruction obtained in the Charpy test

Material	Mechanical properties - Impact strength (Charpy Test)		
	Impact strength (kJ/m^2)		Type of fracture
	Average	SD[a]	
M1	17.1	1.2	Complete
M2	18.4	1.6	Complete
M3	22.7	1.6	Complete
M4	18.9	1.4	Complete
M5	25.7	4.2	Complete
M6	30.2	3.3	Complete
M7	43.0	7.7	Complete
M8	47.7	9.1	Complete
M9	45.5	8.9	Complete
M10	72.4	6.1	Complete
M11	73.3	3.3	Complete
M12	19.5	0.6	Complete
M13	39.1	11	Complete
M14	63.4	4.5	Complete
M15	52.3	5.9	Complete
M16	64.4	9.3	Complete
M17	198	7.4	**Partial**
M18	62.7	2.4	Complete
M19	90.5	5.9	Complete

Note: [a] SD – standard deviation

Table S5. Flexural strength values for selected compositions after accelerated hydrolytic studies[a] (water, 65°C).

Time (days)	Flexural strength (MPa)			
	M9 serie - without HS		M13 serie - with HS	
	Average	SD[b]	Average	SD[b]
0	113.05	11.37	125.43	3.59
7	42.91	1.18	102.74	5.34
14	0.27	0.12	45.61	9.75

Note: [a] accelerated hydrolytic conditions were as follows: samples immersed in water at 65°C; [b]SD – standard deviation

Table S6. Summary of parameters obtained using DSC analysis of the materials.

Material	Thermal properties - DSC			
	T_g (°C)	T_m (°C)	X_{max}[a] (%)	X_c[b] (%)
M1	64	151	11.5	0.1
M2	64	152	18.5	10.6
M3	64	152	22.9	20.2
M4	-30<br>64	151	30.9	21.0
M5	5<br>64	153	26.7	8.1
M6	3<br>62	153	24.2	13.9
M7	63	152	25.1	5.3
M8	5<br>63	151	29.2	9.7
M9	63	153	25.1	8.6
M10	64	157	31.2	18.1
M11	65	157	35.1	29.3
M12	64	152	17.3	1.7
M13	58	154	1.1	0.9
M14	64	154	37.0	32.7
M15	64	157	37.6	31.6
M16	-30<br>59	154	3.0	0.0
M17	-32<br>64	160	40.3	40.3
M18	6<br>65	158	49.6	49.6
M19	-28<br>3<br>62	161	*nd*[c]	*nd*[c]

Note:[a] maximum degree of crystallization obtained under conditions of the experiment, calculated according to the equation (1) in the main text; [b] degree of crystallization of the samples subjected to the experiment, calculated according to the equation (2) in the main text; [c] *nd* - not detected

Table S7. Summary of parameters obtained using TGA analysis of the materials.

Material	Thermal properties - TGA							
	$T_{5\%}$ (°C)	$T_{10\%}$ (°C)	T_{max1} (°C)	T_{max2} (°C)	T_{max3} (°C)	T_{max4} (°C)	T_{max5} (°C)	Weight loss (%)
PBAT	350	371	347	407	570	675	-	100
PHBV	246	249	256	426	652	-	-	100
M1	327	339	369	-	-	-	-	100
M2	328	340	371	-	-	-	-	100
M3	334	342	372	-	-	-	-	100
M4	288	296	313	394	-	-	-	100
M5	286	294	326	418	-	-	-	100
M6	303	310	340	422	-	-	-	100
M7	294	308	346	664	-	-	-	95
M8	297	310	347	645	-	-	-	95
M9	289	303	345	670	-	-	-	94
M10	324	336	370	-	-	-	-	96
M11	322	332	367	-	-	-	-	90
M12	261	275	323	-	-	-	-	100
M13	257	277	273	348	669	-	-	95
M14	334	341	370	-	-	-	-	95
M15	334	342	367	-	-	-	-	93
M16	251	269	274	332	387	547	680	94
M17	332	340	363	-	-	-	-	88
M18	295	303	297	365	-	-	-	92
M19	292	299	300	360	-	-	-	91

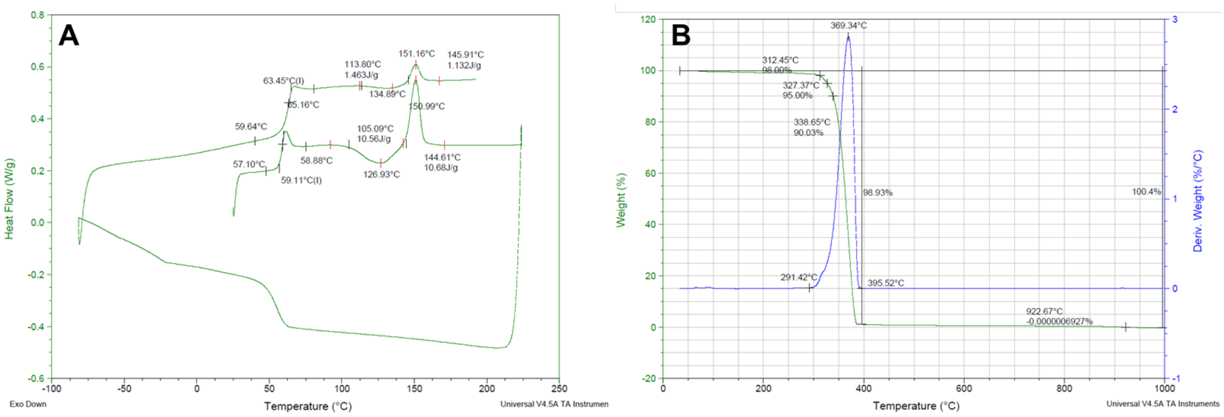

Figure S13. Thermal analysis of sample M1: DSC curve (A) and TGA curve (B).

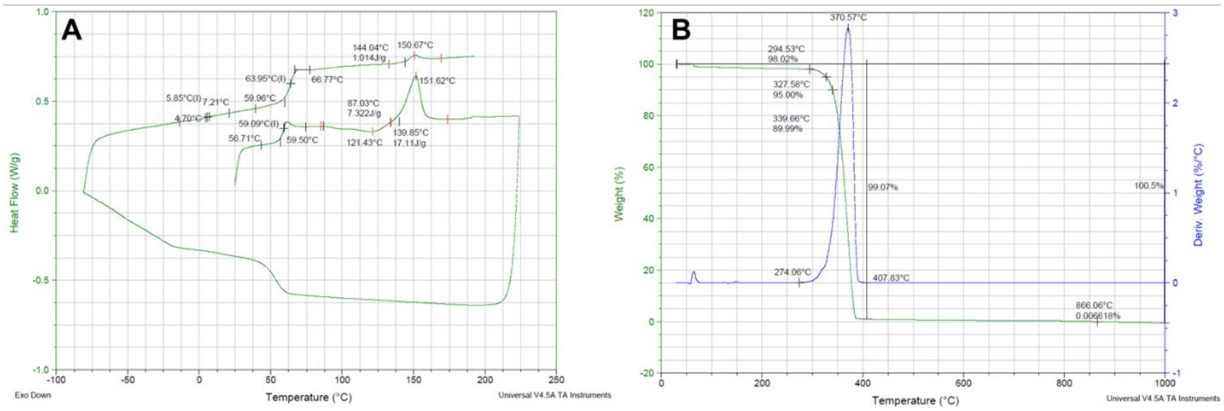

Figure S14. Thermal analysis of sample M2: DSC curve (A) and TGA curve (B).

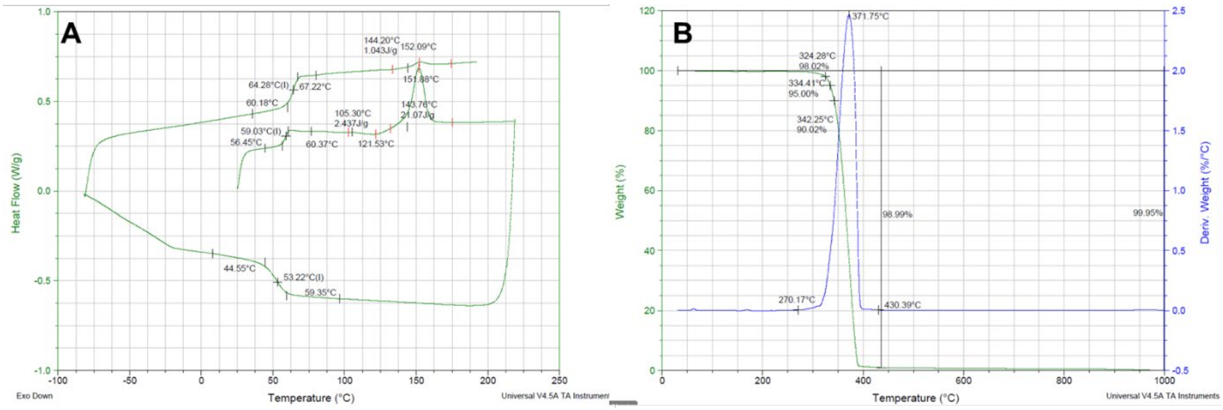

Figure S15. Thermal analysis of sample M3: DSC curve (A) and TGA curve (B).

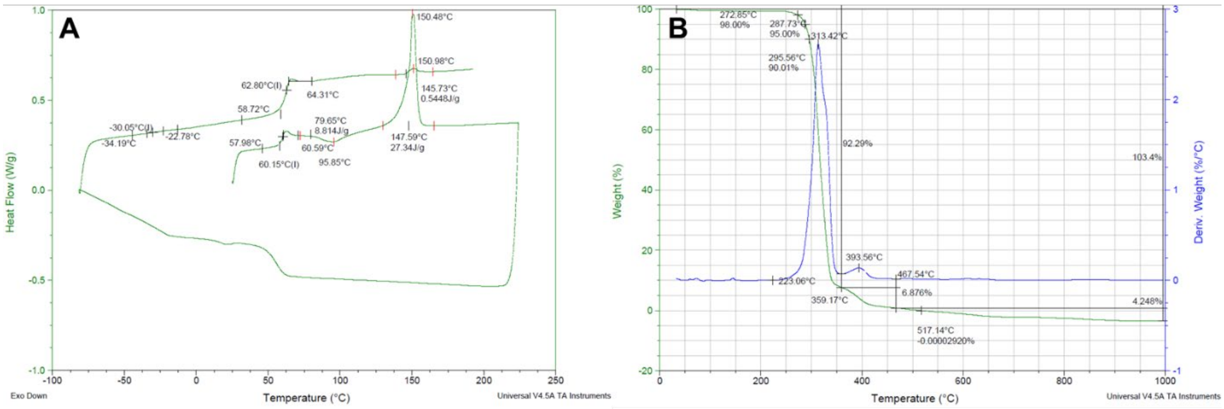

Figure S16. Thermal analysis of sample M4: DSC curve (A) and TGA curve (B).

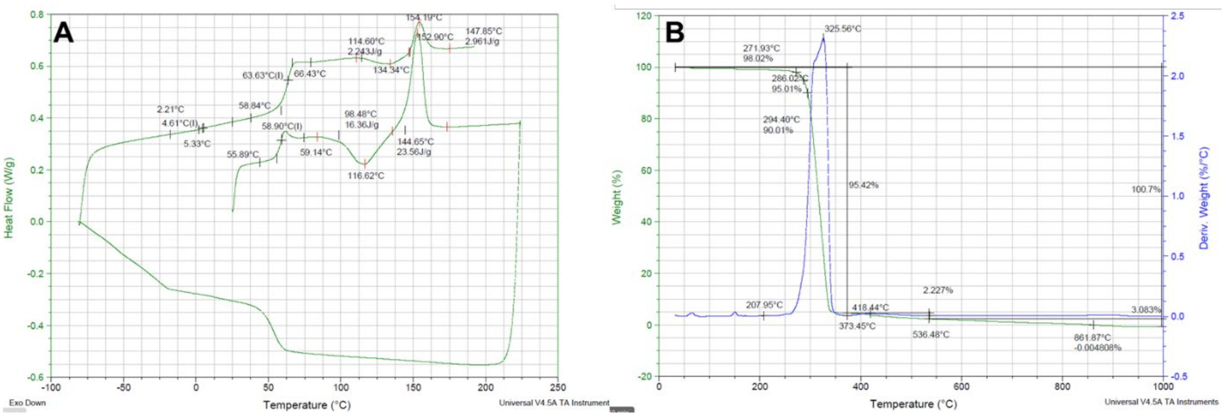

Figure S17. Thermal analysis of sample M5: DSC curve (A) and TGA curve (B).

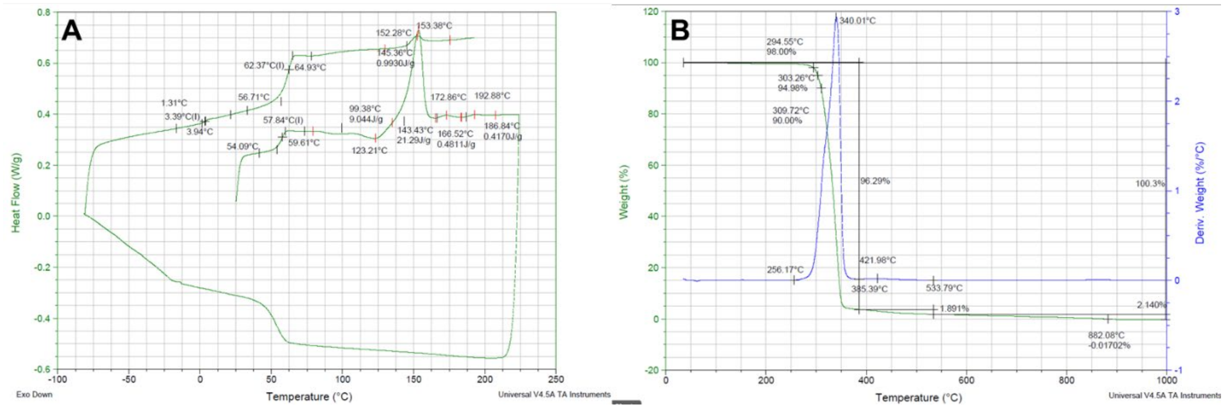

Figure S18. Thermal analysis of sample M6: DSC curve (A) and TGA curve (B).

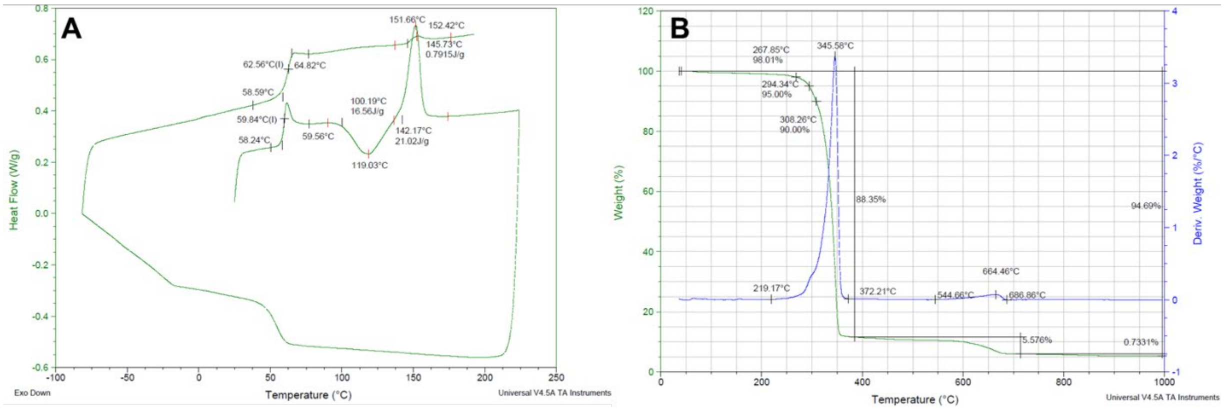

Figure S19. Thermal analysis of sample M7: DSC curve (A) and TGA curve (B).

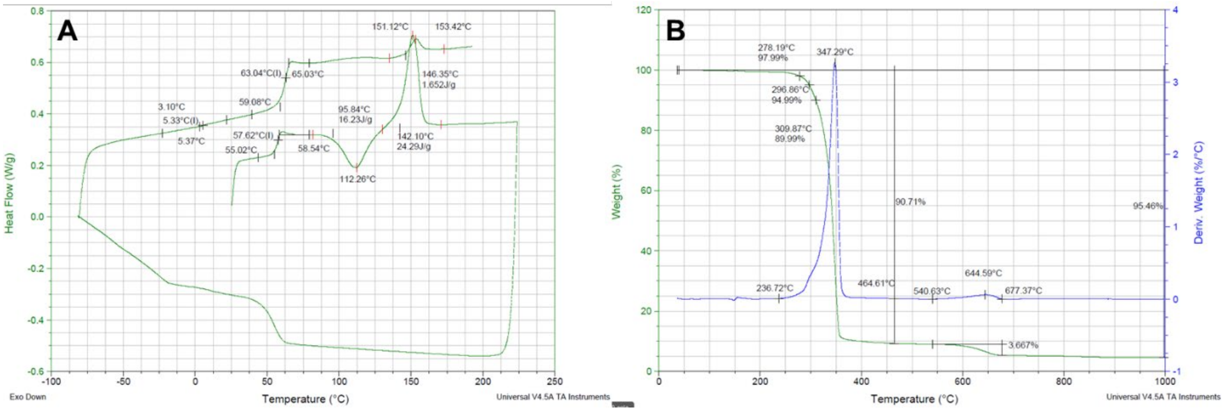

Figure S20. Thermal analysis of sample M8: DSC curve (A) and TGA curve (B).

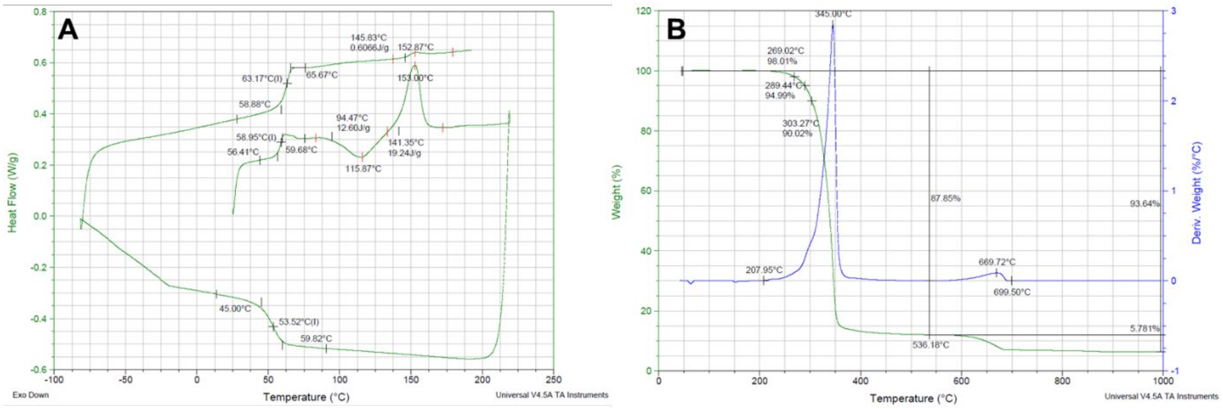

Figure S21. Thermal analysis of sample M9: DSC curve (A) and TGA curve (B).

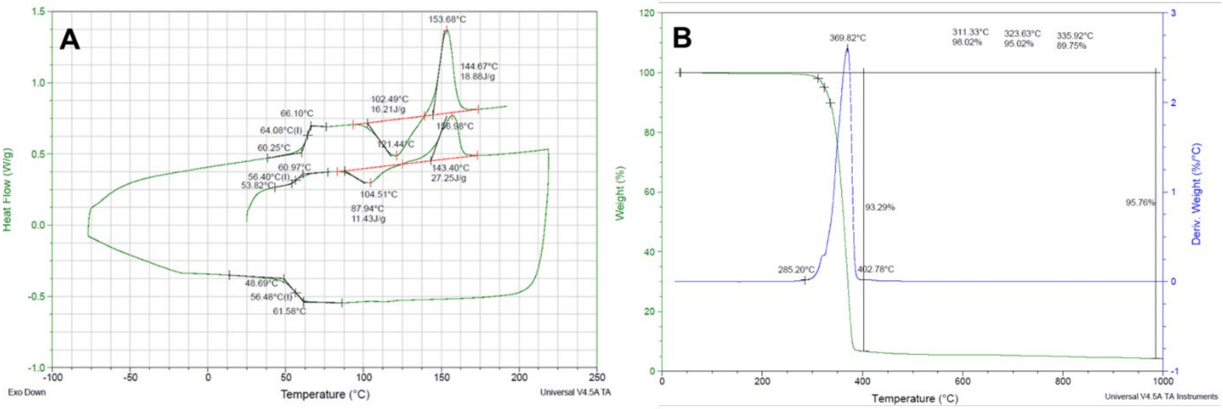

Figure S22. Thermal analysis of sample M10: DSC curve (A) and TGA curve (B).

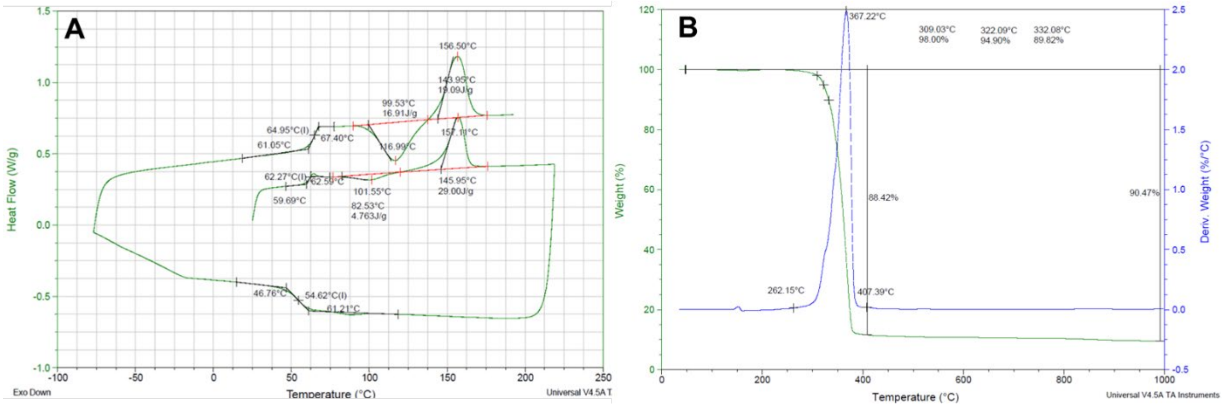

Figure S23. Thermal analysis of sample M11: DSC curve (A) and TGA curve (B).

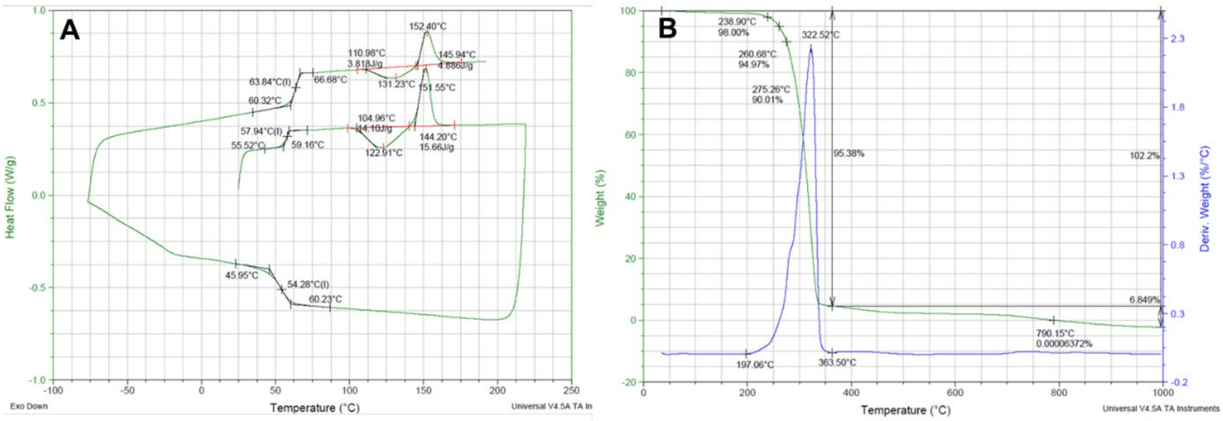

Figure S24. Thermal analysis of sample M12: DSC curve (A) and TGA curve (B).

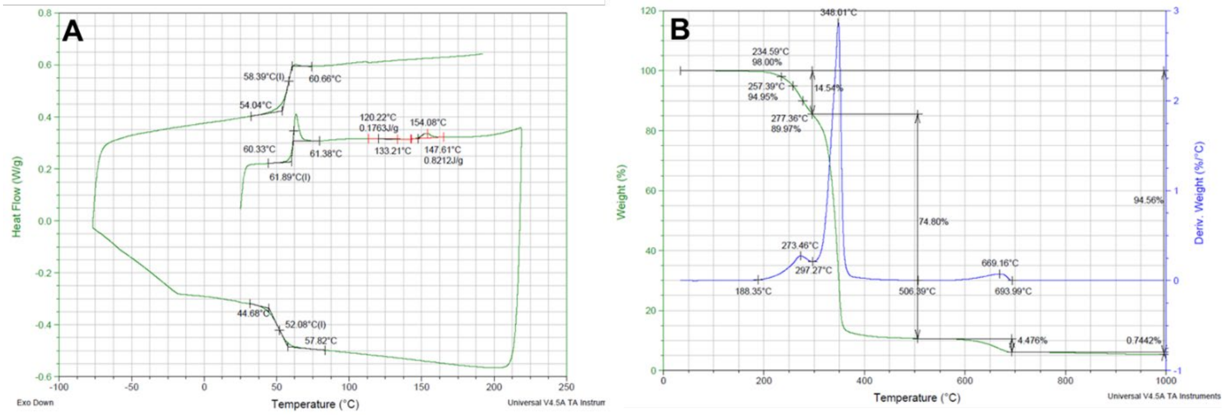

Figure S25. Thermal analysis of sample M13: DSC curve (A) and TGA curve (B).

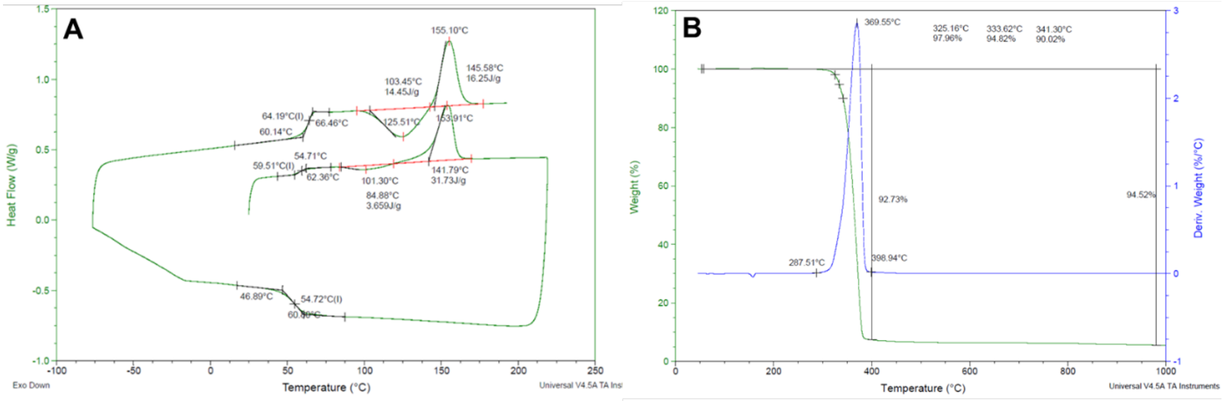

Figure S26. Thermal analysis of sample M14: DSC curve (A) and TGA curve (B).

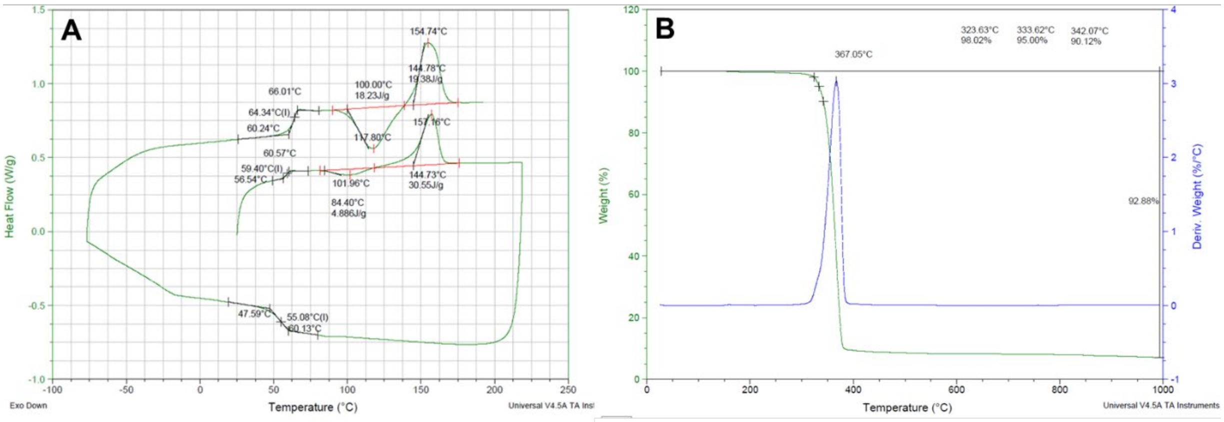

Figure S27. Thermal analysis of sample M15: DSC curve (A) and TGA curve (B).

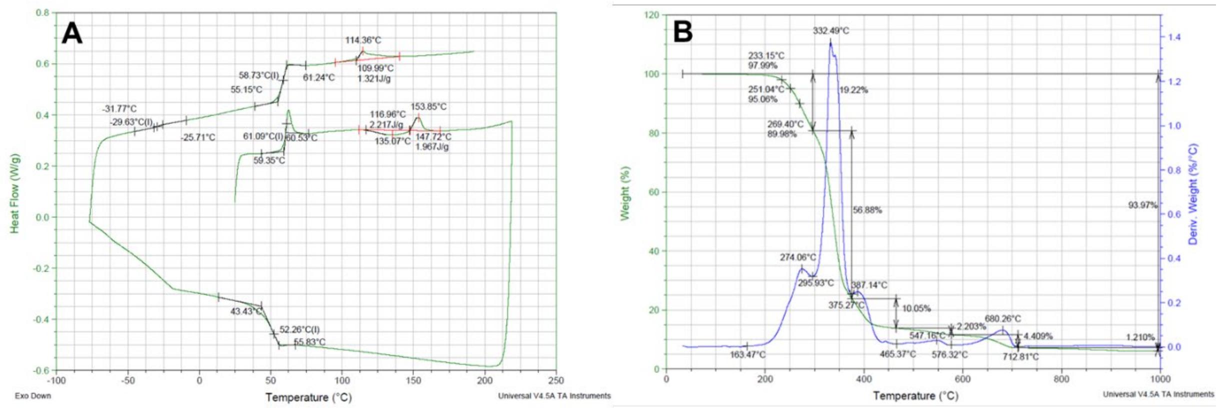

Figure S28. Thermal analysis of sample M16: DSC curve (A) and TGA curve (B).

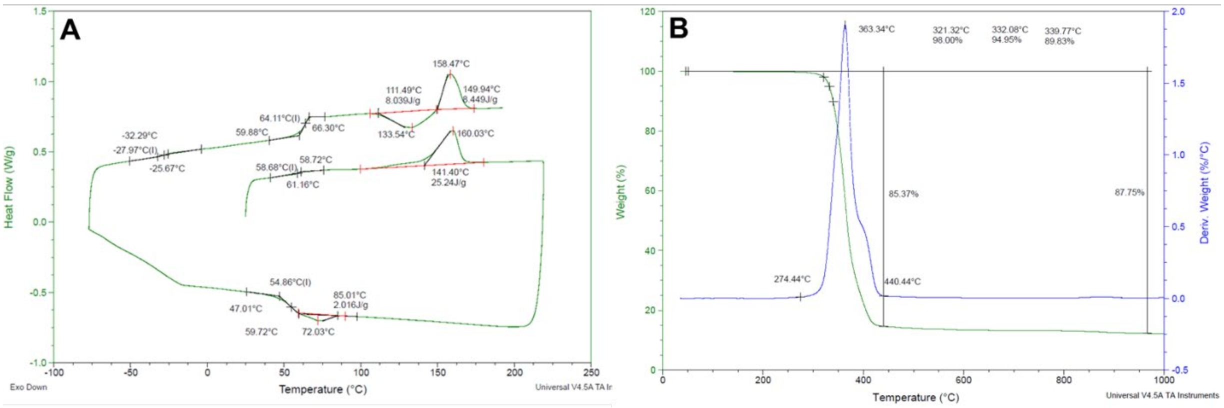

Figure S29. Thermal analysis of sample M17: DSC curve (A) and TGA curve (B).

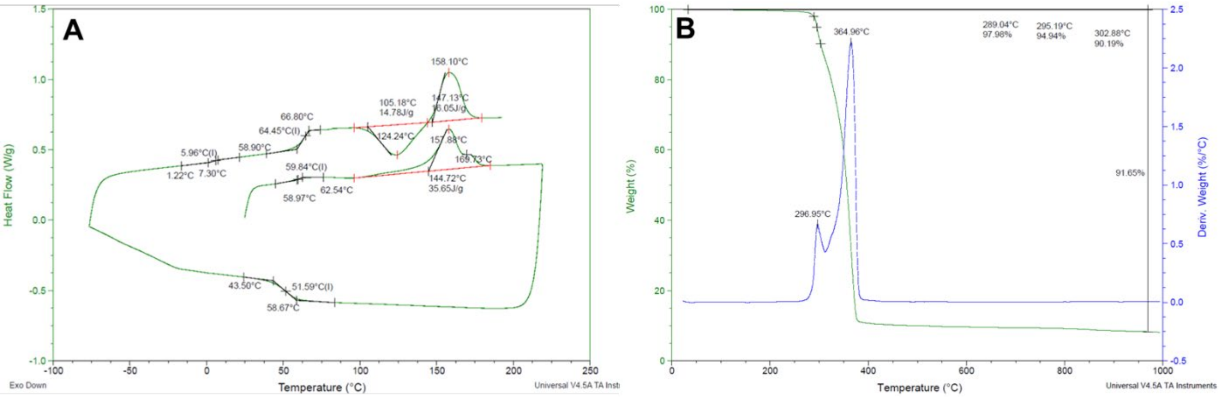

Figure S30. Thermal analysis of sample M18: DSC curve (A) and TGA curve (B).

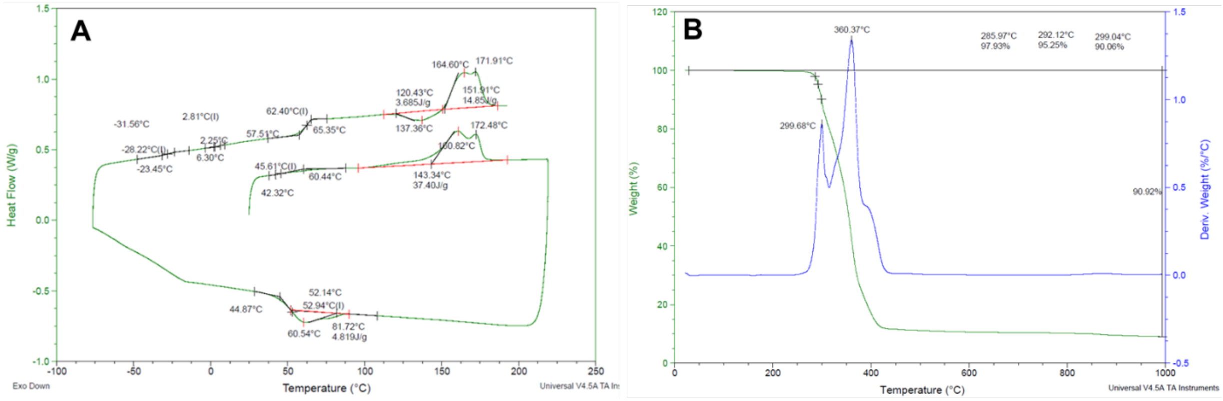

Figure S31. Thermal analysis of sample M19: DSC curve (A) and TGA curve (B).

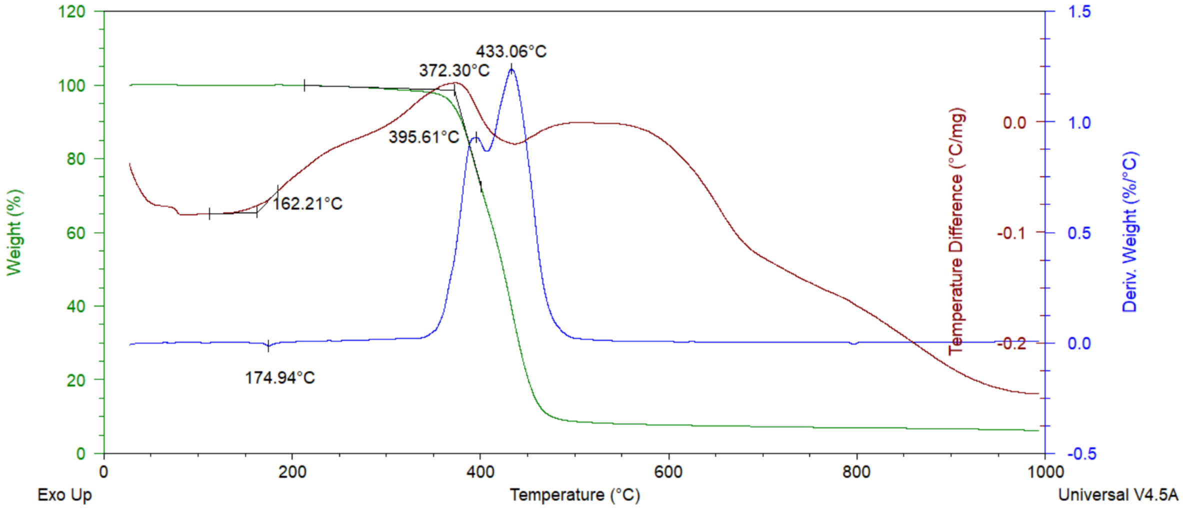

Figure S32. Thermogravimetric analysis of HS.

Table S8. Summary of the Melt Flow Index (MFR) results of obtained polymer mixtures.

Material	Melt Flow Index MFR at 190 °C, 2.16 kg (g · 10 min^{-1})
M1	4.9
M2	1.5
M3	0.4
M4	12.0
M5	7.2
M6	2.0
M7	17.1
M8	11.2
M9	6.9
M10	3.4
M11	1.8
M12	1.6
M13	8.6
M14	1.6
M15	2.1
M16	9.5
M17	1.3
M18	1.9
M19	1.8

Table S9. SEM images of compositions M1-M19 (as-manufactured surfaces and fractures).

<table>
<tr><td rowspan="4">M1</td><td>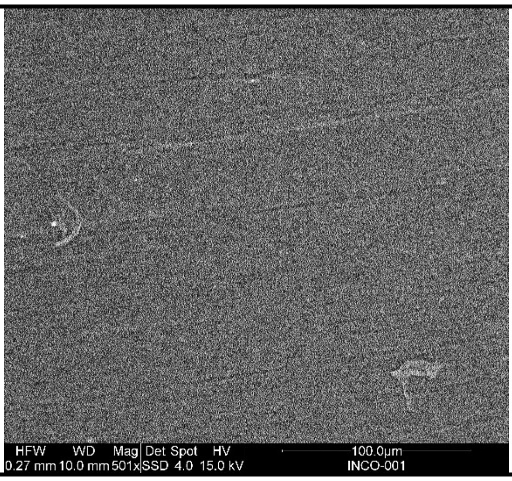
</td><td>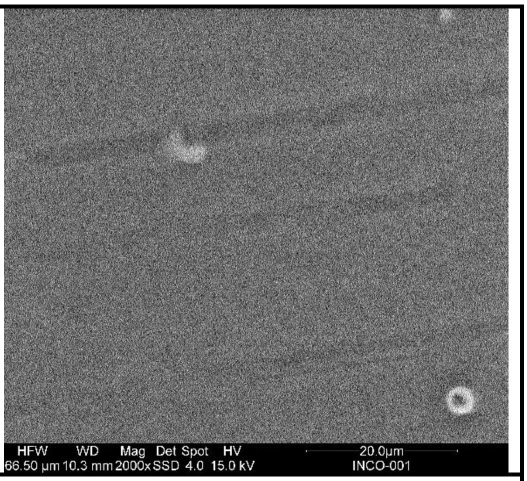
</td></tr>
<tr><td>100 µm surface</td><td>20 µm surface</td></tr>
<tr><td>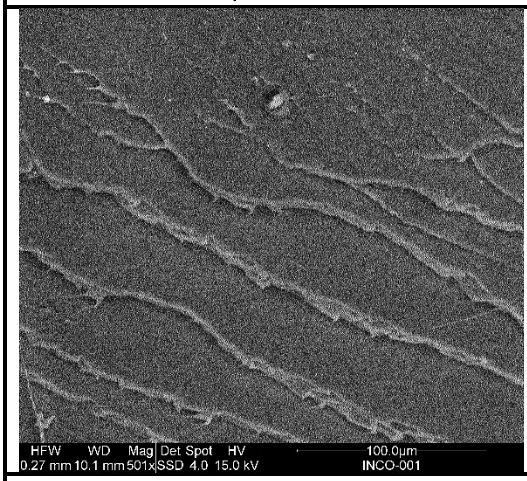
</td><td>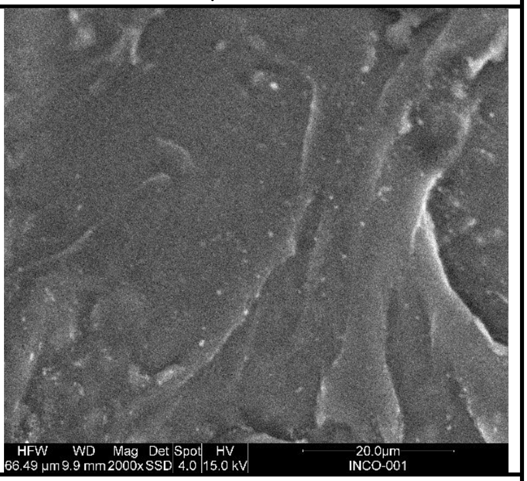
</td></tr>
<tr><td>100 µm fracture</td><td>20 µm fracture</td></tr>
</table>

<table>
<tr><td rowspan="4">M2</td><td>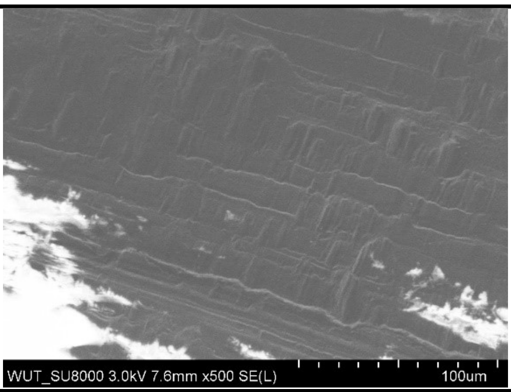
</td><td>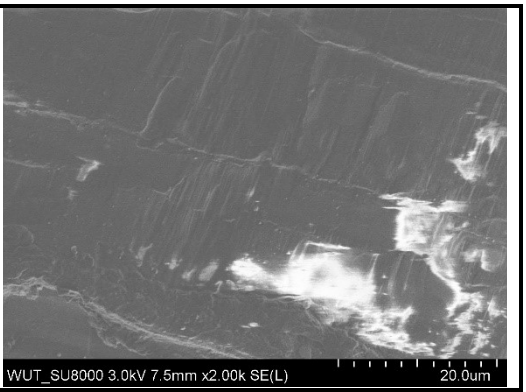
</td></tr>
<tr><td>100 μm surface</td><td>20 μm surface</td></tr>
<tr><td>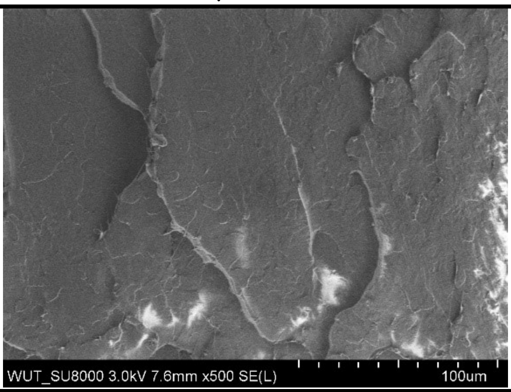
</td><td>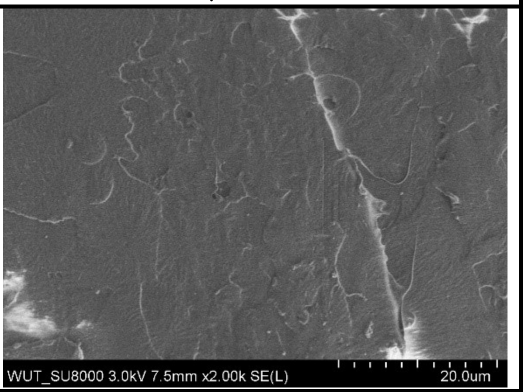
</td></tr>
<tr><td>100 μm fracture</td><td>20 μm fracture</td></tr>
<tr><td rowspan="4">M3</td><td>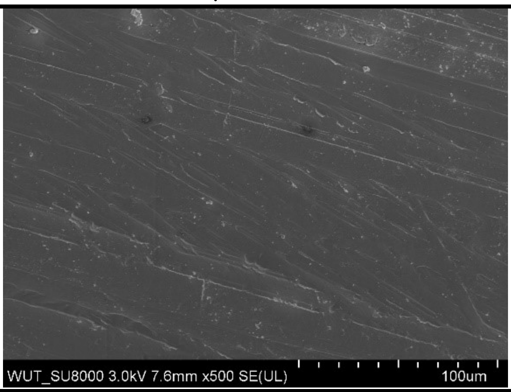
</td><td>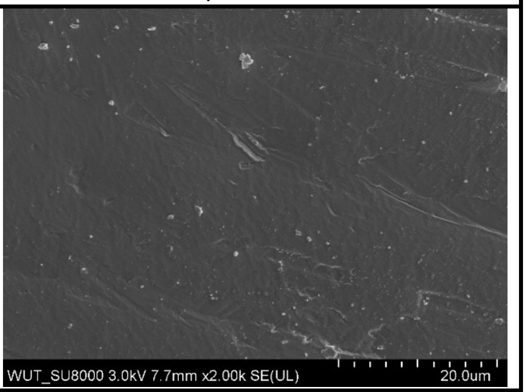
</td></tr>
<tr><td>100 μm surface</td><td>20 μm surface</td></tr>
<tr><td>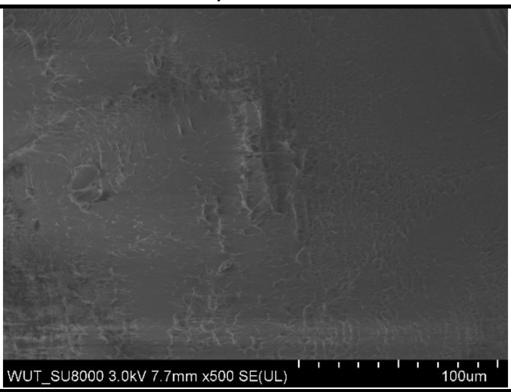
</td><td>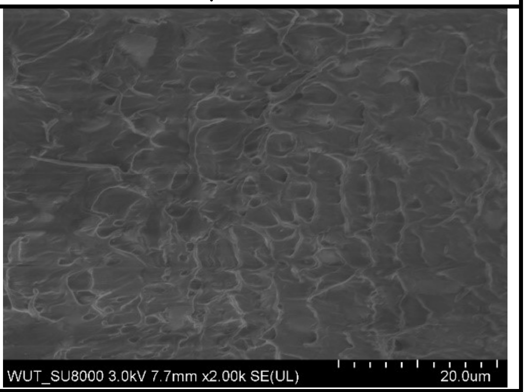
</td></tr>
<tr><td>100 μm fracture</td><td>20 μm fracture</td></tr>
</table>

M4	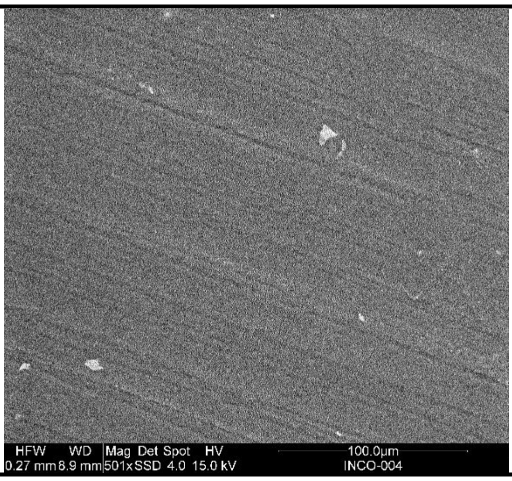	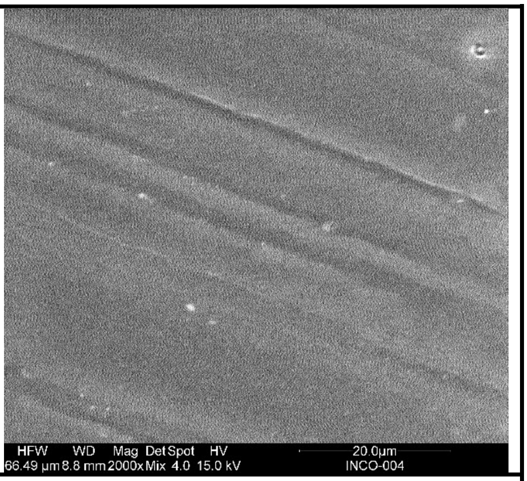
	100 µm surface	20 µm surface
	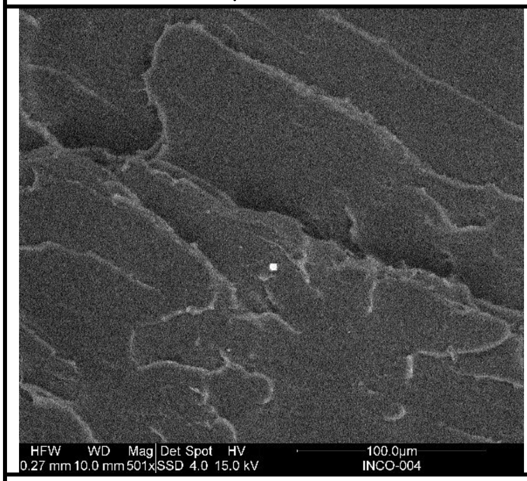	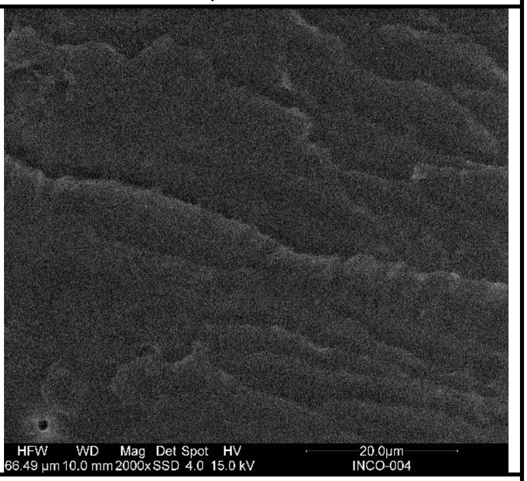
	100 µm fracture	20 µm fracture
M5	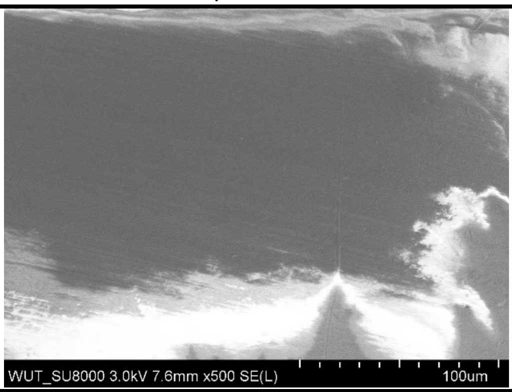	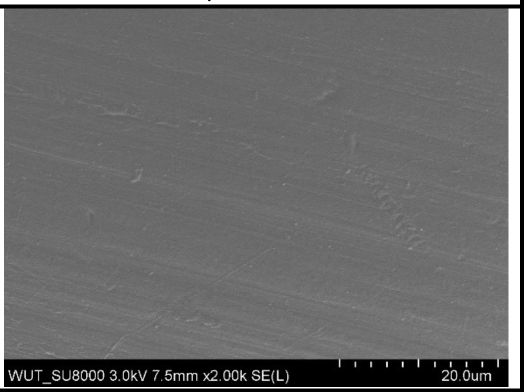
	100 µm surface	20 µm surface
	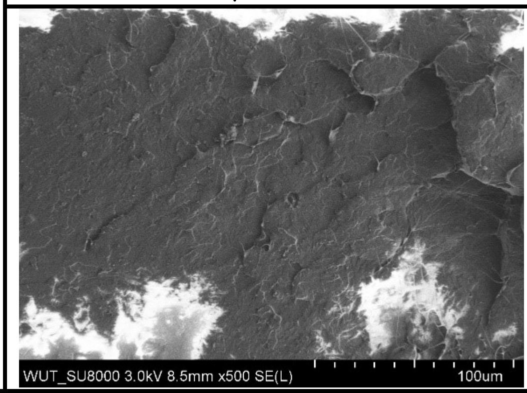	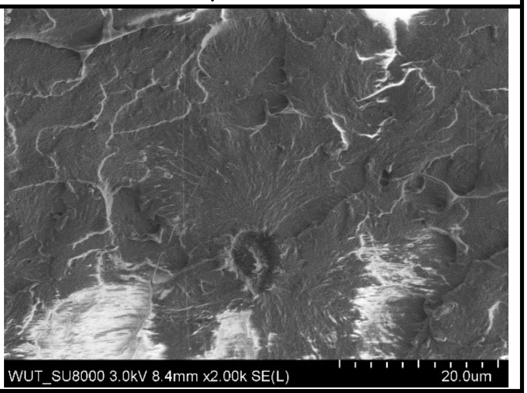

<table>
<tr><td></td><td>100 µm fracture</td><td>20 µm fracture</td></tr>
<tr><td rowspan="4">M6</td><td>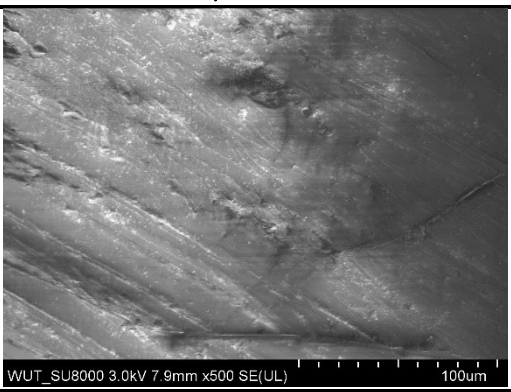
</td><td>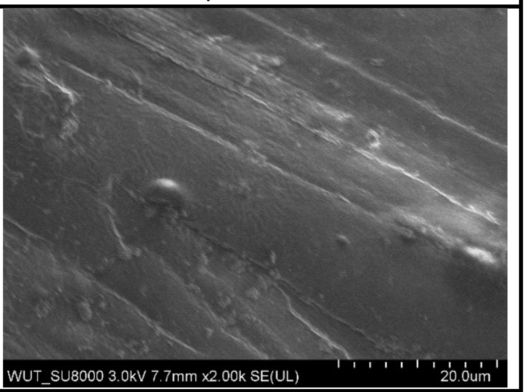
</td></tr>
<tr><td>100 µm surface</td><td>20 µm surface</td></tr>
<tr><td>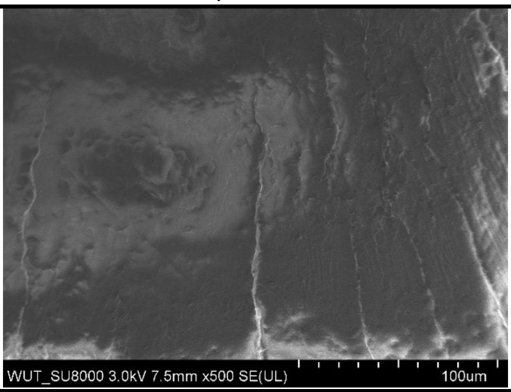
</td><td>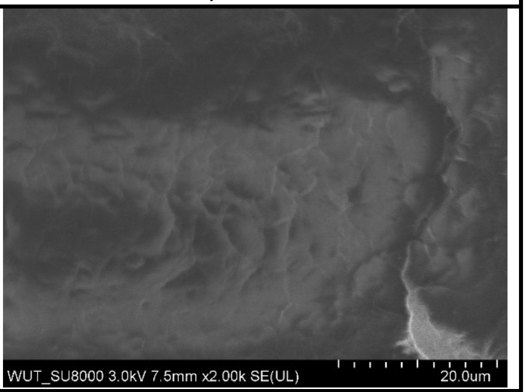
</td></tr>
<tr><td>100 µm fracture</td><td>20 µm fracture</td></tr>
<tr><td rowspan="4">M7</td><td>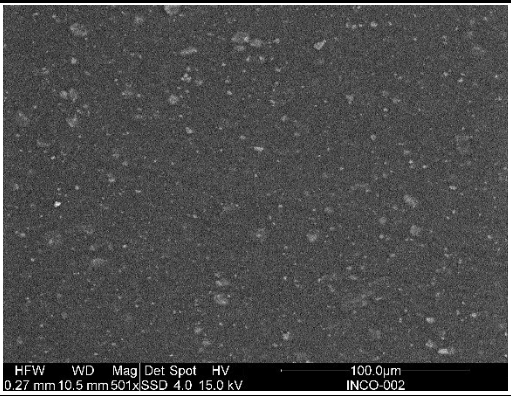
</td><td>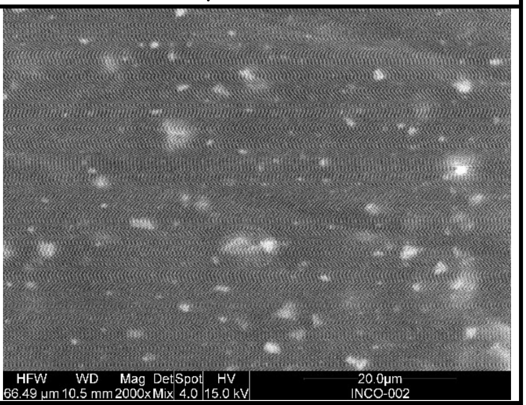
</td></tr>
<tr><td>100 µm surface</td><td>20 µm surface</td></tr>
<tr><td>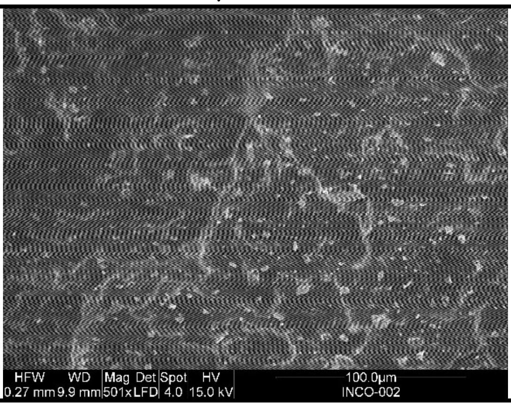
</td><td>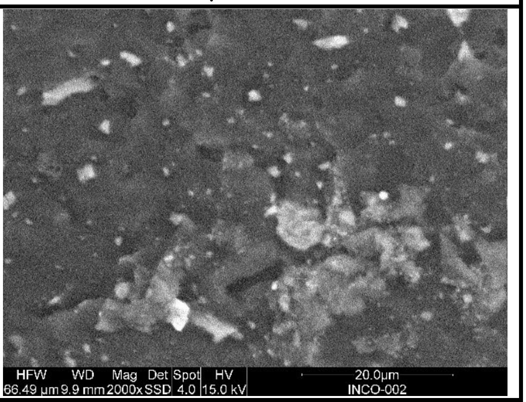
</td></tr>
<tr><td>100 µm fracture</td><td>20 µm fracture</td></tr>
</table>

M8

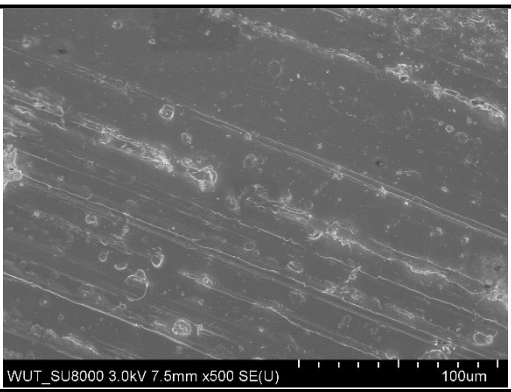

100 μm surface

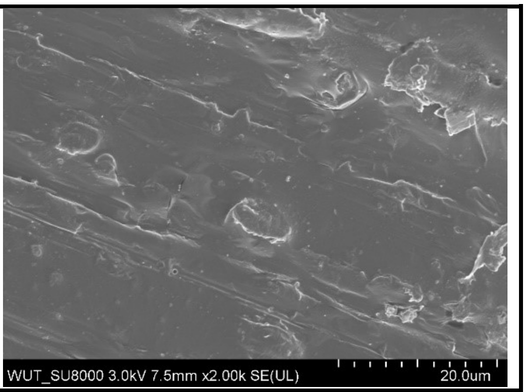

20 μm surface

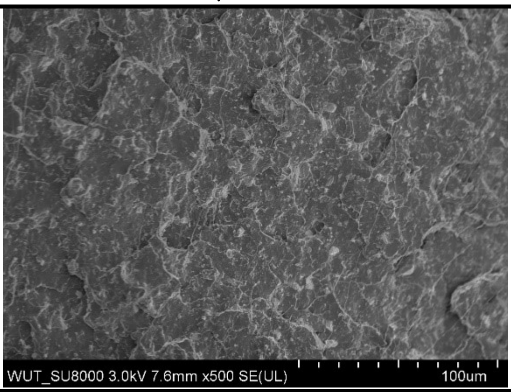

100 μm fracture

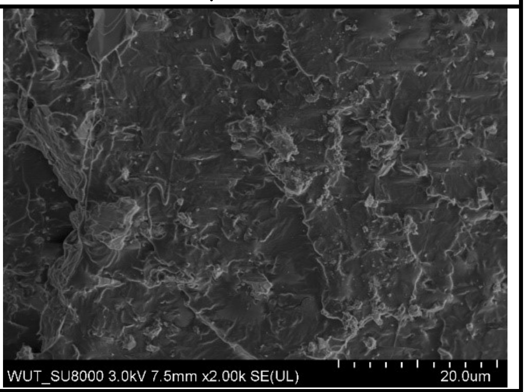

20 μm fracture

M9

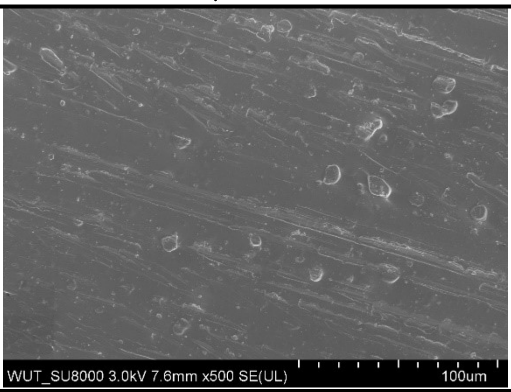

100 μm surface

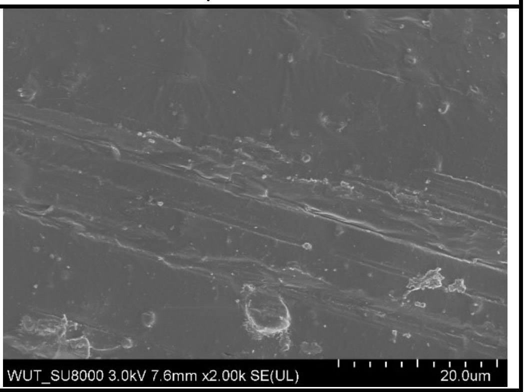

20 μm surface

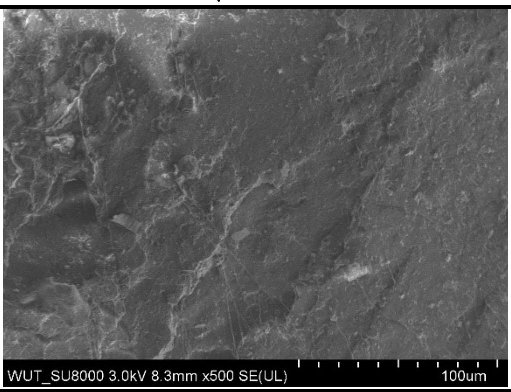

100 μm fracture

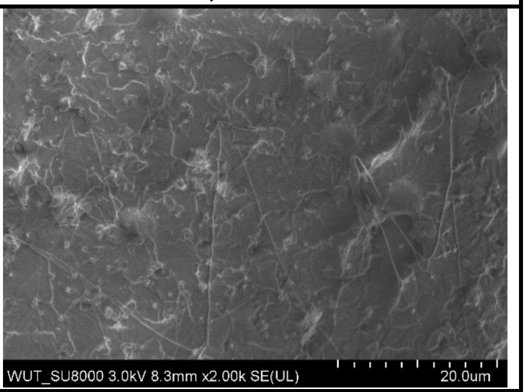

20 μm fracture

<table>
<tr><td rowspan="4">M10</td><td>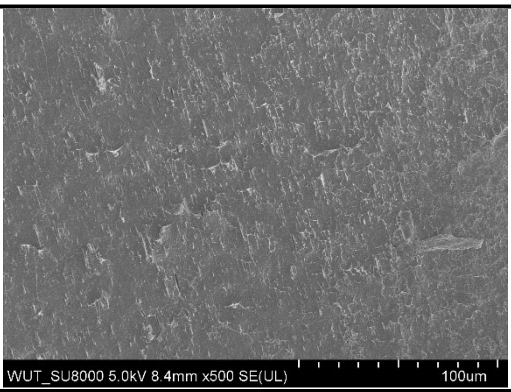
</td><td>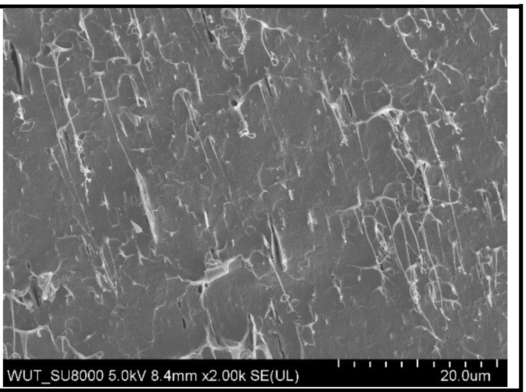
</td></tr>
<tr><td>100 μm surface</td><td>20 μm surface</td></tr>
<tr><td>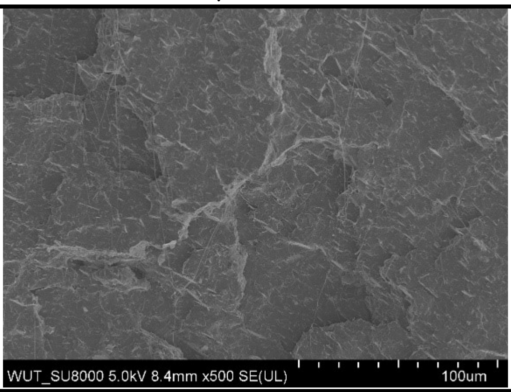
</td><td>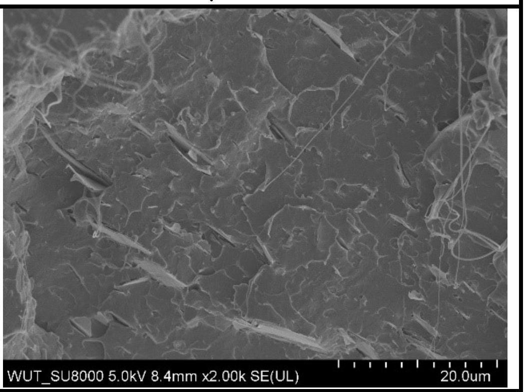
</td></tr>
<tr><td>100 μm fracture</td><td>20 μm fracture</td></tr>
<tr><td rowspan="4">M11</td><td>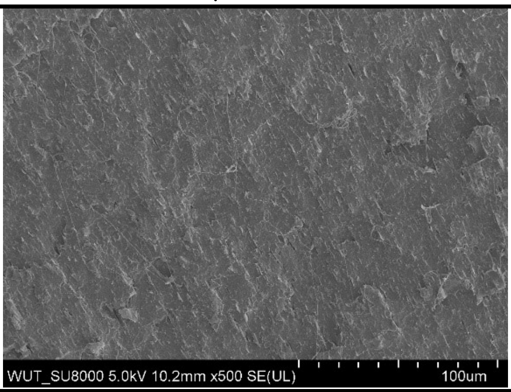
</td><td>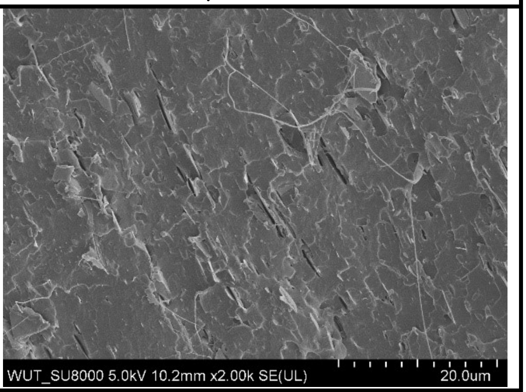
</td></tr>
<tr><td>100 μm surface</td><td>20 μm surface</td></tr>
<tr><td>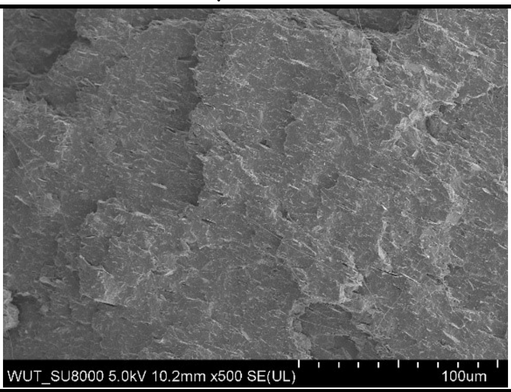
</td><td>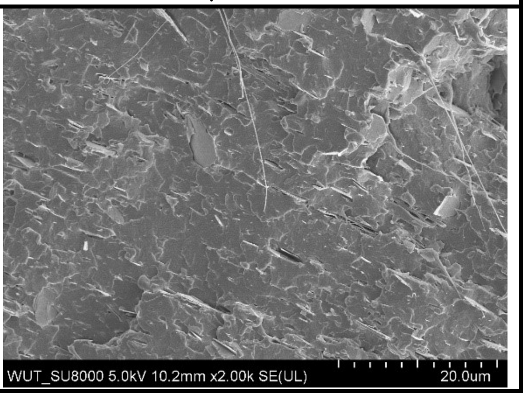
</td></tr>
<tr><td>100 μm fracture</td><td>20 μm fracture</td></tr>
</table>

<table>
<tr><td rowspan="2">M12</td><td>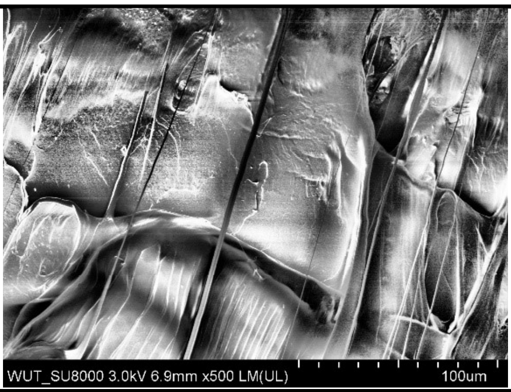
</td><td>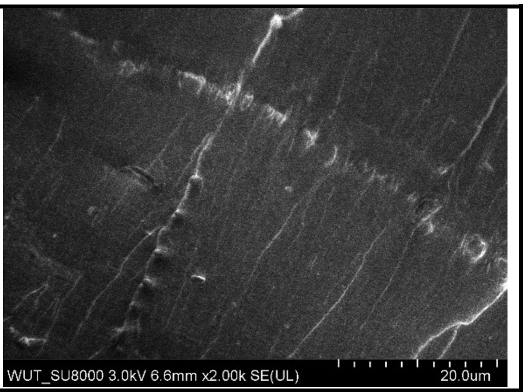
</td></tr>
<tr><td>100 μm surface</td><td>20 μm surface</td></tr>
<tr><td rowspan="2">M13</td><td>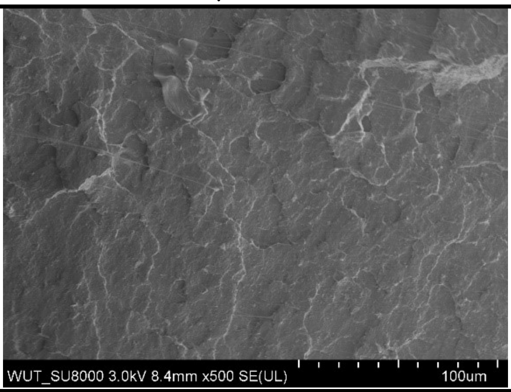
</td><td>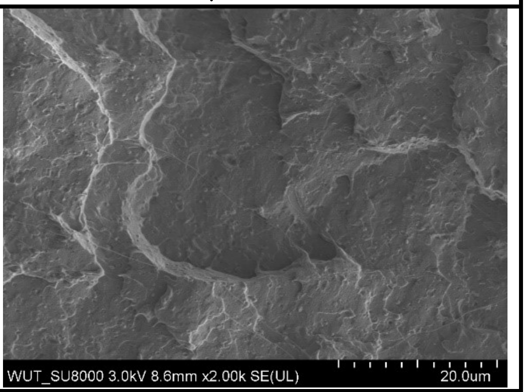
</td></tr>
<tr><td>100 μm fracture</td><td>20 μm fracture</td></tr>
<tr><td rowspan="4">M14</td><td>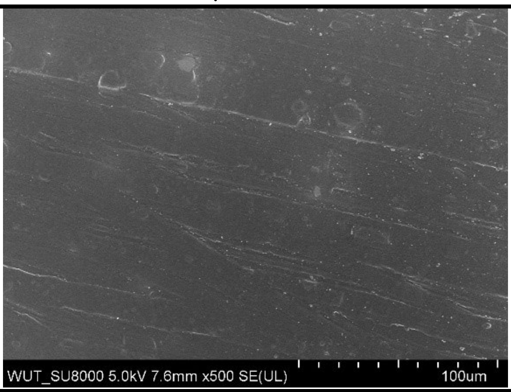
</td><td>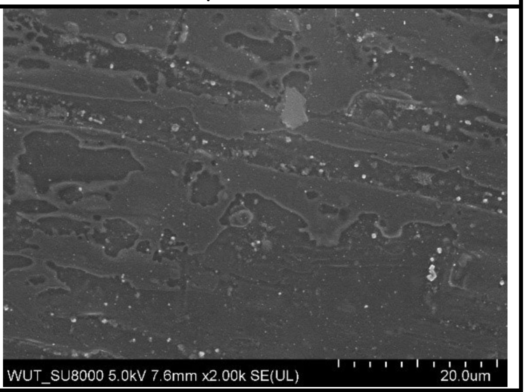
</td></tr>
<tr><td>100 μm surface</td><td>20 μm surface</td></tr>
<tr><td>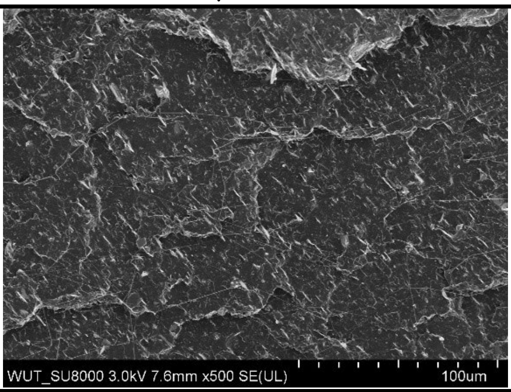
</td><td>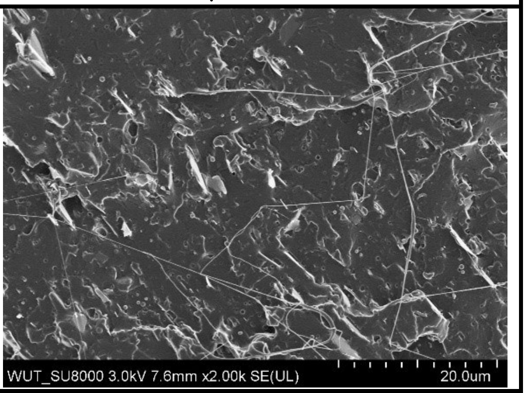
</td></tr>
<tr><td>100 μm fracture</td><td>20 μm fracture</td></tr>
</table>

<table>
<tr><td rowspan="4">M15</td><td>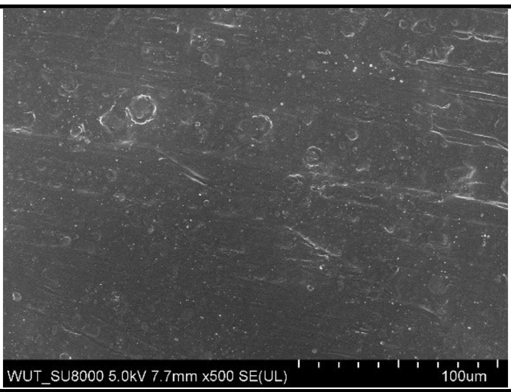
</td><td>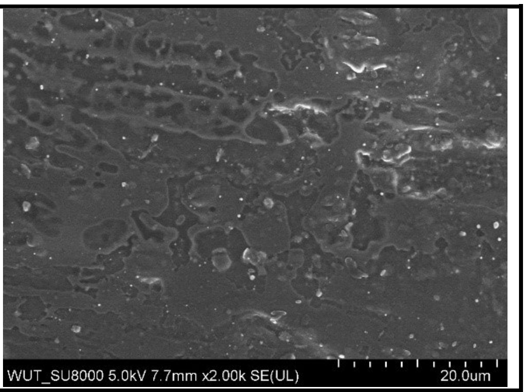
</td></tr>
<tr><td>100 μm surface</td><td>20 μm surface</td></tr>
<tr><td>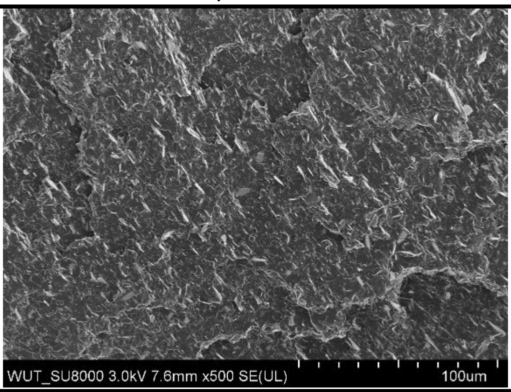
</td><td>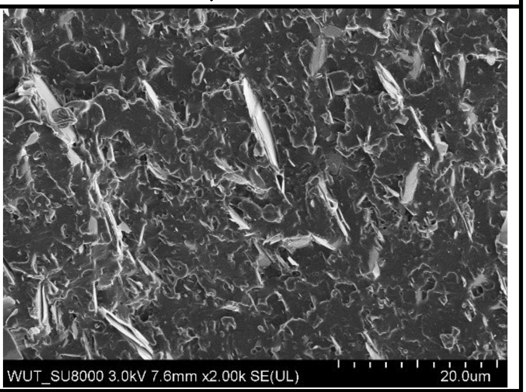
</td></tr>
<tr><td>100 μm fracture</td><td>20 μm fracture</td></tr>
<tr><td rowspan="2">M16</td><td>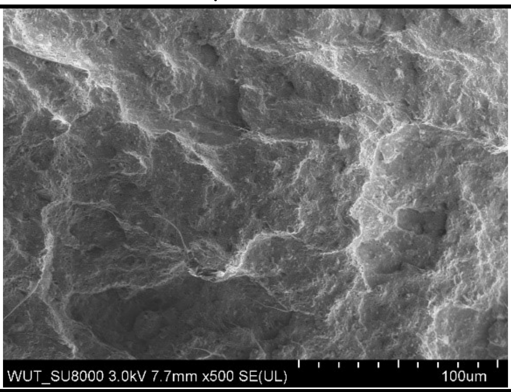
</td><td>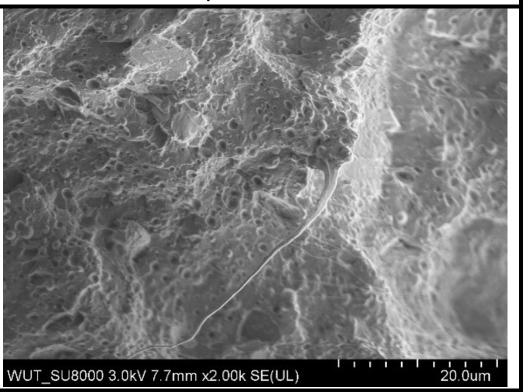
</td></tr>
<tr><td>100 μm fracture</td><td>20 μm fracture</td></tr>
</table>

<table>
<tr><td rowspan="4">M17</td><td>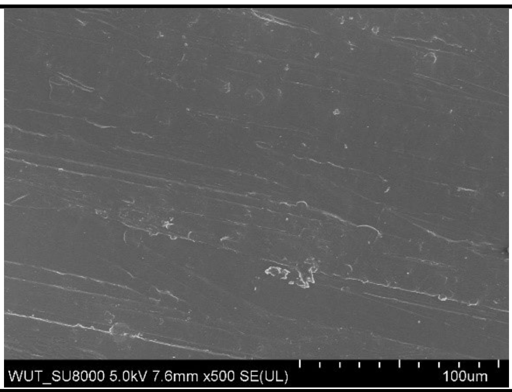
</td><td>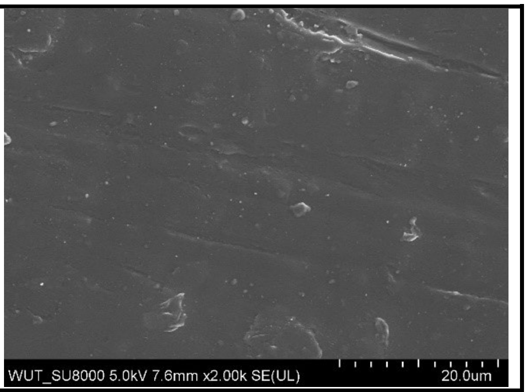
</td></tr>
<tr><td>100 μm surface</td><td>20 μm surface</td></tr>
<tr><td>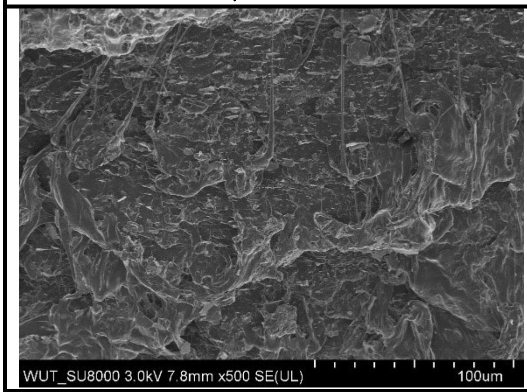
</td><td>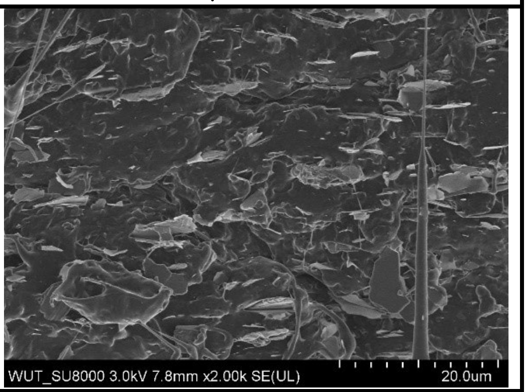
</td></tr>
<tr><td>100 μm fracture</td><td>20 μm fracture</td></tr>
<tr><td rowspan="4">M18</td><td>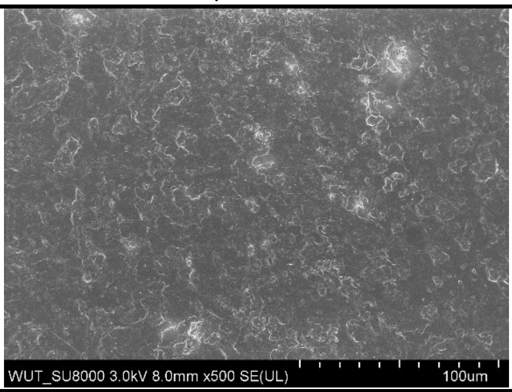
</td><td>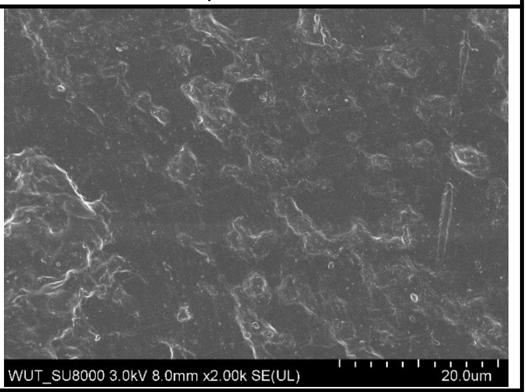
</td></tr>
<tr><td>100 μm surface</td><td>20 μm surface</td></tr>
<tr><td>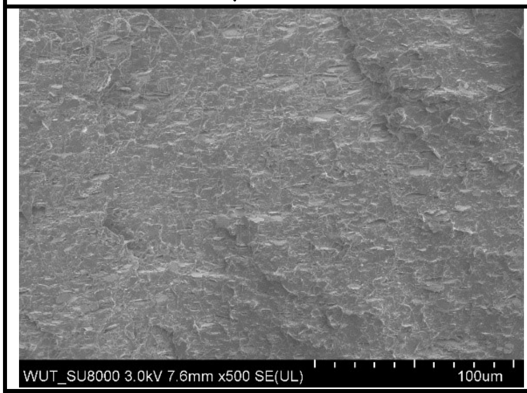
</td><td>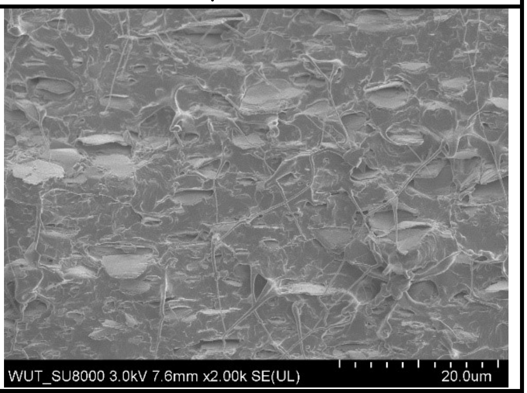
</td></tr>
<tr><td>100 μm fracture</td><td>20 μm fracture</td></tr>
</table>

<table>
<tr><td rowspan="4">M19</td><td>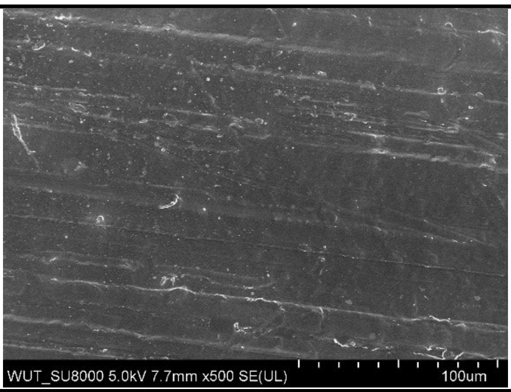
</td><td>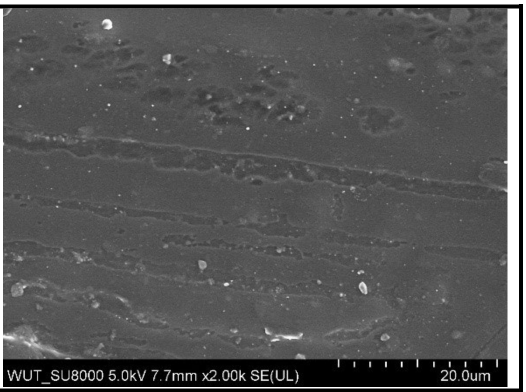
</td></tr>
<tr><td>100 µm surface</td><td>20 µm surface</td></tr>
<tr><td>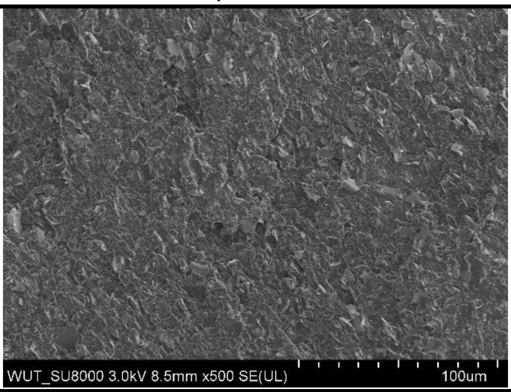
</td><td>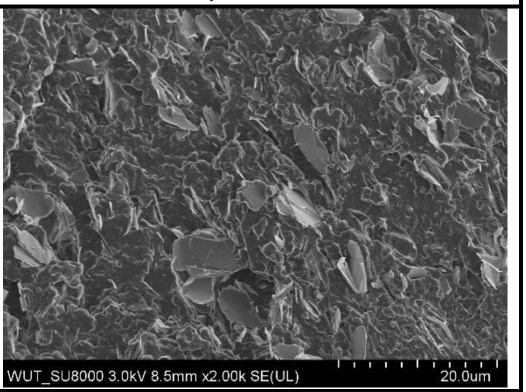
</td></tr>
<tr><td>100 µm fracture</td><td>20 µm fracture</td></tr>
</table>

The data, including raw GPC analysis reports and an Excel spreadsheet with statistical tests of the strength data, are available in the "WUT Research Data Repository."

File link: **DOI: 10.71724/maj4-rh44**
